# AMPA receptors and their minions: auxiliary proteins in AMPA receptor trafficking

**DOI:** 10.1007/s00018-019-03068-7

**Published:** 2019-04-01

**Authors:** Diane Bissen, Franziska Foss, Amparo Acker-Palmer

**Affiliations:** 1 Institute of Cell Biology and Neuroscience and Buchmann Institute for Molecular Life Sciences (BMLS), University of Frankfurt, Max-von-Laue-Str. 15, 60438 Frankfurt am Main, Germany; 2 Max Planck Institute for Brain Research, Max von Laue Str. 4, 60438 Frankfurt am Main, Germany; 3 Cardio-Pulmonary Institute (CPI), Max-von-Laue-Str. 15, 60438 Frankfurt am Main, Germany

**Keywords:** AMPA receptors, Trafficking, Synapse, GRIP1, PICK1, MAGUK, TARP, CNIH

## Abstract

To correctly transfer information, neuronal networks need to continuously adjust their synaptic strength to extrinsic stimuli. This ability, termed synaptic plasticity, is at the heart of their function and is, thus, tightly regulated. In glutamatergic neurons, synaptic strength is controlled by the number and function of AMPA receptors at the postsynapse, which mediate most of the fast excitatory transmission in the central nervous system. Their trafficking to, at, and from the synapse, is, therefore, a key mechanism underlying synaptic plasticity. Intensive research over the last 20 years has revealed the increasing importance of interacting proteins, which accompany AMPA receptors throughout their lifetime and help to refine the temporal and spatial modulation of their trafficking and function. In this review, we discuss the current knowledge about the roles of key partners in regulating AMPA receptor trafficking and focus especially on the movement between the intracellular, extrasynaptic, and synaptic pools. We examine their involvement not only in basal synaptic function, but also in Hebbian and homeostatic plasticity. Included in our review are well-established AMPA receptor interactants such as GRIP1 and PICK1, the classical auxiliary subunits TARP and CNIH, and the newest additions to AMPA receptor native complexes.

## Introduction

Synaptic plasticity is a core feature of neuronal networks and describes their ability to adjust the strength of their connections in response to extrinsic stimuli. It is, therefore, highly regulated at both ends of the synaptic cleft. On the postsynaptic side, neuronal sensitivity is regulated by adapting the number and properties of available receptors at the membrane. When these changes are long-lasting, they are referred to as long-term potentiation (LTP) or long-term depression (LTD), depending on whether the synaptic strength is increased or decreased, respectively. This type of plasticity is collectively called Hebbian plasticity, underlies learning and memory, and represents one of the first brain functions to suffer in neurodegenerative diseases [1, 2]. Moreover, neurons are also able to sense their own activity levels and to return to their baseline, thereby ensuring a certain stability in the network. This process, called homeostatic synaptic scaling, also depends on the insertion or removal of receptors from the membrane—insertion increases neuronal sensitivity to neurotransmitters (scaling up), whereas removal decreases it (scaling down) [3].

Glutamate is the major excitatory neurotransmitter in the central nervous system, and its major ionotropic receptors are the *N*-methyl-d-aspartate (NMDA) and α-amino-3-hydroxy-5-methyl-4-isoxazolepropionic acid (AMPA) receptors. NMDA receptors (NMDARs) are both ligand- and voltage-gated: their activation depends not only on the binding of glutamate, but also on the concomitant depolarization of the postsynaptic membrane following neuronal activity, which relieves the block of their ion channel by magnesium. AMPA receptors (AMPARs), on the other hand, are ligand-gated only and the primary mediators of fast excitatory transmission. The dynamic regulation of AMPARs trafficking to, at, and from the synaptic membrane is a key aspect of synaptic plasticity [4]. From their assembly onwards as homo- or heterotetramers of four highly homologous subunits GluA1-4, AMPARs are, however, never alone but surrounded by a multitude of proteins throughout their lifetime, which guide their subcellular destination and fate. The role of these interacting proteins has attracted more and more attention over the past few years, as it became clearer that they were playing a major role in regulating AMPAR trafficking and function. Their temporally and spatially regulated expression, leading to different combinations according to age, brain region, neuronal type, and even cellular localization, also provides a molecular framework underlying the spatio-temporal specific features of AMPAR trafficking ([5]; reviewed in Ref. [6]). For instance, mutant mice lacking the interacting protein transmembrane AMPAR regulatory protein (TARP) γ2 or γ8 present defects in their major region of expression—the cerebellum or hippocampus, respectively [7, 8]. Similarly, pharmacological companies select now interacting proteins as drug targets, as their restricted expression pattern allows for a more specific modulation of receptor signaling (reviewed in Ref. [9]).

In addition to their timing and location, the strength and stability of the interaction between the auxiliary proteins and AMPARs is of course variable. Some of them were found repeatedly in proteomics studies, and have, therefore, been classified as parts of native AMPAR macrocomplexes. Those include an “inner core”, composed of the strongest bound proteins [TARPs, cornichon proteins (CNIHs), and germline-specific gene 1-like (GSG1L)], and an “outer core” of peripheral, more variable content [10]. Interestingly, not all of the historically well-known AMPAR interacting proteins, such as glutamate receptor-interacting protein (GRIP) 1 and protein interacting with C kinase (PICK) 1, were identified in proteomic studies. These diverging results may, of course, arise from technical differences between the protocols, which were designed for different purposes (stringency of the interaction, confirmation of single partners versus unbiased protein screening). More interestingly, highly dynamic interactions and/or subunit specificity might also be the reason for the lack of some interactors in those studies [11, 12]. This could apply very well to GRIP1 and PICK1, which also act as scaffolds for larger complexes.

This review will focus on the major direct interacting partners of AMPA receptors regulating their trafficking. Due to the impossibility of describing the variety of roles undertaken by these proteins, we will refer our readers to recent reviews regarding their role in AMPAR complex assembly, gating, and function, but also AMPAR trafficking specifically at the endoplasmic reticulum (ER) and during aging and diseases [13–17]. In this review, we will give an overview of the complex network of interactions surrounding AMPARs, concentrating on the role of AMPAR partners in regulating AMPAR trafficking in mature neurons, especially at the synapse. We will start with proteins containing PDZ [Postsynaptic density (PSD) 95, *Drosophila* discs large homolog (Dlg) 1, and zonula-occludens 1 protein (zo1)] domains, i.e., GRIP1, PICK1, and the MAGUK (membrane-associated guanylate kinase) family; we will continue with the typical auxiliary subunits TARP and CNIH, and finish with the partners newly identified by Schwenk and colleagues.

## PDZ domain-containing interactants

### Glutamate receptor-interacting proteins (GRIPs)

**Fig. 1:**
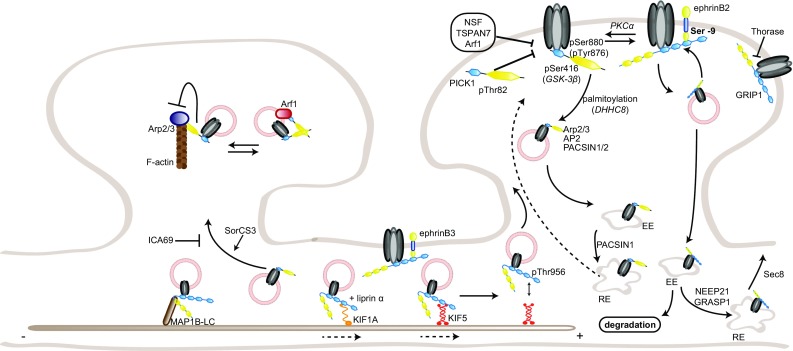
Regulation of AMPAR trafficking by GRIP1 and PICK1. The PDZ domain-containing GRIP1 and PICK1 regulate surface AMPAR in opposite directions: GRIP1 primarily promotes AMPAR surface insertion, stabilization, and reinsertion after internalization, while PICK1 acts on internalization and intracellular anchorage. Their interaction with GluA2 subunits is regulated by GluA2 phosphorylation on the serine 880, which favors GluA2–PICK1 binding over GluA2–GRIP1. GRIP1 interaction with ephrinB ligands stabilizes surface AMPARs, at spine (ephrinB2, via the phosphorylation of ephrinB2 serine-9) or shaft (ephrinB3) synapses. Following internalization, PICK1–GluA2 also inhibits actin polymerization. PICK1 regulates AMPAR sorting, while GRIP1 also transports AMPARs to the synapse by interacting with the motor proteins KIF1A and KIF5; cargo release from KIF5 and the microtubules requires phosphorylation of GRIP1–Thr956 residue. Conversely, GRIP1 interaction with MAP1B-LC leads to AMPAR–GRIP1 trapping on the microtubules. *EE* early endosome, *RE* recycling endosome. Note for all figures: posttranslational modifications (with the causative enzymes, when known) and direct partners of AMPAR interactants relevant for their regulation of AMPAR trafficking are also included. No stoichiometry is implicated; binding sites are indicative only, and size ratios are not to scale

AMPAR subunits GluA2 and GluA3 share a common sequence (-SVKI) at the end of their C-terminus, through which they can interact with PDZ domain-containing proteins [18]. Two groups have been identified so far: the GRIP family of proteins [18, 19] and PICK1 [20]. GRIP proteins include the original member GRIP1 [18], as well as GRIP2 [21, 22] and AMPAR-binding protein (ABP; [19]). GRIP1/2 contains seven PDZ domains, while ABP, a shorter splice variant of GRIP2, lacks the seventh PDZ domain [22]. The role of GRIP proteins in regulating AMPAR trafficking to and at the synapse has been intensively studied. GRIP1, but not GRIP2, binds to the motor protein kinesin superfamily protein (KIF) 5 and acts as a cargo for AMPARs and other proteins, such as erythropoietin-producing human hepatocellular receptor (EphB) receptors and N-cadherin, transporting them into the dendrites [23–27] (Fig. 1). GRIP1 release from KIF5 is regulated by the phosphorylation of the threonine residue 956 of GRIP1, which subsequently binds another partner, the 14-3-3 proteins; this is an important step for the function of AMPARs at the synapse. Mutating this threonine to an alanine, and thereby preventing its phosphorylation, impairs GRIP1 function; phosphodeficient mice show impaired cargo trafficking, but also decreased dendritogenesis [25]. This phosphorylation is carried by the kinase Akt1 [27].

At the synapse, GRIP1 role is more ambiguous. Several studies have, indeed, shown GRIP1 requirement in AMPAR insertion to the synaptic surface [28–36], while others have linked it to AMPAR removal from the synaptic membrane and intracellular anchorage [37–39]. On one hand, GRIP1 binding to GluA2 is regulated by GluA2 phosphorylation on the serine 880 (pSer880) by protein kinase C (PKC), which decreases GluA2 affinity for GRIP1, but does not affect its interaction with PICK1 [29, 40]. pSer880 has, therefore, been suggested as a “switch” regulating GluA2 interaction with both PDZ-containing proteins. Upon Ser880 phosphorylation, GluA2 release from GRIP1 is associated with its internalization and the dissociation of its postsynaptic clusters [32], and its subsequent binding to PICK1 is required for the maintenance of cerebellar LTD [33]. A similar mechanism has been proposed for hippocampal LTD, suggesting a complementary role for GRIP1 and PICK1 in regulating the insertion and internalization of AMPARs at the surface, respectively [29, 31, 34]. Consistently, GluA2 phosphorylation on Ser880 is required for cerebellar LTD [30]. In addition, GRIP1/2 double knockout mice show increased pSer880- GluA2 levels and decreased surface AMPARs, while transgenic mice with gain-of-function mutations display enhanced surface distribution and faster recycling of AMPARs [41, 42]. On the other hand, GRIP1 has also been shown to bind internalized GluA2 receptors and tether them intracellularly following LTD induction or AMPA-induced endocytosis, suggesting a role in preventing AMPAR reinsertion at the membrane [37, 39, 43]. A similar role has been proposed for GRIP2 [44]. In agreement with this, GRIP1/2 double knockout Purkinje cells are unable to express LTD [45]. In addition to AMPAR insertion or internalization, GRIP1 may also regulate activity-dependent AMPAR reinsertion into the membrane, by interacting with the exocyst protein Sec8 [46]. To add a further level of complexity, PICK1 is also able to bind the second linker domain of GRIP proteins; interfering with such interaction decreases Ser880 phosphorylation and surface GluA2 expression, NMDAR-induced GluA2 internalization and its subsequent reinsertion into the membrane, suggesting a regulatory role for PICK1/GRIP1 interaction as well [47]. Finally, GluA2 phosphorylation on the tyrosine 876 also regulates its binding to PDZ-containing proteins similarly to Ser880 phosphorylation, as it decreases GRIP1/2 binding without affecting PICK1 interaction; mutating this residue leads to a reduction of GluA2 receptors at the membrane, further emphasizing the main role for GRIP1 in regulating surface expression of AMPARs [48].

All these functions are, however, not mutually exclusive, if one considers the existence of multiple AMPAR pools at the synapse, with different recycling rates, and possibly involving different regulatory proteins and responding to different stimuli. Indeed, not all GluA2-containing receptors are internalized upon Ser880 phosphorylation by PKC; a subpopulation stays inserted at the membrane, suggesting that binding to GRIP1 is not required by all the receptors for surface expression [49]. Moreover, triggering NMDAR- or mGluR-dependent LTD in hippocampal neurons leads to the endocytosis of different AMPAR populations: while the former induces internalization of rapidly recycling AMPARs, which are not bound to GRIP, the latter triggers the endocytosis of stable, GRIP-bound AMPARs [50].

Recently, a new function of GRIP1 in homeostatic synaptic scaling has also been uncovered [35, 36]. Using either shRNA against GRIP1 or GRIP1/2 double knockout neurons, two studies showed that GluA2–GRIP1 interaction is required for the trafficking and insertion of AMPARs in the membrane during scaling up following silencing of neuronal activity; it is, however, not required for scaling down, consistently with a major role of GRIP1 in surface AMPAR insertion rather than internalization [36].

Another level of regulation arises from the existence of several GRIP1/2 isoforms that undergo different modes of posttranslational modification. GRIP1, indeed, has five isoforms to date (GRIP1a–e) and GRIP2 two [38, 51–53]. GRIP1c–e display different subcellular localization and are also found in inhibitory–aminobutyric acid (GABA) expressing synapses, but their specific function is still unknown [52, 53]. GRIP1a/b and the two GRIP2 isoforms differ by an 18 amino acid variant at the extreme N-terminus that can be palmitoylated [38, 51]. Palmitoylation regulates GRIP1a/b intracellular localization and its role in NMDAR-induced AMPAR internalization, which is enhanced by overexpression of palmitoylated GRIP1b, but inhibited by overexpression of unpalmitoylable GRIP1a [54]. In agreement, GRIP1b palmitoylation by DHHC5/8 directs GRIP1b to dendritic endosomes and enhances AMPAR recycling [55]. While GRIP2 colocalizes with internal membranes, palmitoylated GRIP2 (p-GRIP2) is targeted to the plasma membrane in spines, suggesting a differential role as synaptic and intracellular anchoring sites for AMPARs during activity-induced recycling [38]. Overexpressing p-GRIP2 increases surface AMPAR abundance and synaptic transmission, as well as colocalization with N-cadherin [56]. Interestingly, these results suggest a different role of palmitoylation in regulating GRIP functions, as palmitoylation of GRIP2 but not GRIP1 has an effect on basal AMPAR trafficking.

In addition to AMPARs, GRIP1 interacts with several other proteins, including the extracellular matrix protein Fras1, whose mutations cause the Fraser syndrome in human patients. GRIP1 knockout mice show similar defects to Fras1 knockout mice, such as subepidermal hemorrhagy, renal agenesis, and closing of eyelids, and the absence of this interaction is most likely the reason why GRIP1 knockout mice die embryonically [57]. GRIP1 also binds the MAGUK protein calcium/calmodulin-dependent serine protein kinase (CASK), another protein involved in glutamate receptor trafficking [58, 59]. GRIP1 interacts as well with neuron-enriched endosomal protein of 21 kDa (NEEP21), an early endosomal protein which is crucial for proper receptor recycling in neurons [60, 61] and colocalizes with GluA2/3 receptors at the postsynaptic density (PSD) [62]. Overexpression of NEEP21 slows recycling of AMPARs to the dendritic membrane, while depletion decreases surface GluA2 abundance and basal synaptic transmission, and, in turn, blocks LTP, suggesting a role for the GRIP1–NEEP21 interaction in constitutive AMPAR cycling, but also activity-dependent sorting and reinsertion of AMPARs from the endosomes [61, 63] (Fig. 1). GRIP1–NEEP21 binding is regulated by phosphorylation of the serine 917 of GRIP1a; such phosphorylation occurs after the formation of the complex and is likely involved into GRIP1 dissociation from the endosomes to allow insertion of AMPARs into the membrane [64].

GRIP1 interacts as well with the neuron-specific guanine exchange factor GRIP1-associated protein 1 (GRASP1). GRASP1 is part of the complex including GRIP1 and GluA2, and is involved in constitutive and NMDA-induced AMPAR synaptic targeting [65]. Recently, GRASP1 was also involved in LTP maintenance following activation by the translational regulator cytoplasmic polyadenylation element-binding protein 2 (CPEB2; [66]). Of note, GRASP1 synaptic abundance is increased by prenatal cocaine exposure via PKC- and Src-mediated hyperphosphorylation of GRIP1, likely mediating AMPAR dysfunction and dendritic defects observed in cocaine-exposed brains [67, 68]. GRASP1 has, furthermore, been shown to coordinate endosomal recycling by segregating Rab4 and early endosome antigen 1 (EEA1)/NEEP21/Rab5 early endosomes via its binding to the soluble NSF attachment protein receptor (SNARE) protein syntaxin 13, a protein that also interacts with NEEP21, thereby regulating AMPAR recycling to the surface [60, 69]. This regulatory role has not only functional but also morphological consequences, as it is necessary for proper AMPAR recycling, synaptic plasticity, and spine morphology. Consistently, GRASP1 knockout mice display lower glutamatergic synapse density, reduced LTP, and impaired learning-induced AMPAR delivery and cognitive ability. GRASP1 mutations have been now found in human patients with intellectual disability, and these mutations affect AMPAR recycling as well, opening the door to a better understanding of the etiology of such impairments [70]. Interestingly, loss- or gain-of-function of GRIP proteins affects social interactions, and in humans, five missense GRIP1 variants leading to a gain-of-function have also been associated with autism [41, 42].

In addition, GRIP1 scaffolds a complex including the focal adhesion protein liprin α, the leukocyte common antigen-related (LAR) family receptor protein tyrosine phosphatases (LAR-RPTP), and AMPARs, which is enriched at postsynaptic sites. Interfering with the GRIP1–liprin α binding decreases surface expression and clustering of AMPARs [71]. A similar reduction was observed when interfering with the binding of an additional member of the complex, the actin regulatory protein ARF GTPase-activating protein (GIT) 1 [72]. Cadherin and β-catenin may also be included into this complex, whose transport to the dendrite is regulated by the LAR-RPTP, implicating GRIP1 in regulating spine morphology [73]. Interestingly, GRIP1-liprin α interaction has also been involved in muscarinic-induced hippocampal LTP [74]. GRIP1 and liprin α also associate with the motor protein KIF1A [75], suggesting that GRIP1 can interact with more than one motor protein. Indeed, GRIP1 has also been shown to bind the light chain of microtubule-associated protein (MAP) 1B, an interaction required for dihydroxyphenylglycine (DHPG)-induced AMPAR internalization and regulating its trafficking towards the dendrite [76, 77].

Another partner of GRIP1 is the AAA + ATPase Thorase, or ATPase family AAA domain-containing 1 (Atad1), which regulates in an ATP-dependent fashion AMPAR internalization by disassembling GRIP1–GluA2 complexes (Fig. 1). Upon Thorase depletion, AMPAR internalization and LTD are consequently decreased, while LTP is enhanced [78]. Thorase-deficient mice display disrupted AMPAR internalization and recycling and behavioral deficits, which can be rescued by the FDA-approved drug Perampanel [79]. Interestingly, Thorase variants have been found in schizophrenia patients and Perampanel use in patients improved hypertonicity and resolution of seizures, showing a promising start [80]. Very recently, additional Thorase mutants were found in patients with lethal encephalopathy. In mice, those mutations result in a gain-of-function that affects expression levels of multiple proteins as well as the disassembly of GRIP1–GluA2 complexes, leading to a decreased surface GluA2 expression [81]. Altogether, these studies directly involve GRIP1 in the etiology of several human diseases, underlying the importance of understanding its function in AMPAR trafficking.

Last but not least, GRIP1 also interacts with the Eph family of receptor tyrosine kinases and their ephrin ligands; more precisely, with ephrinB ligands, their cognate receptor EphB2, but also EphA7 [82]. The Eph/ephrin family of signaling molecules displays a bidirectional mode of signaling, as binding of ephrin ligands to Eph receptors triggers signaling downstream of Eph (forward signaling), but also of ephrin (reverse signaling). In addition to their crucial roles in multiple processes during brain development, the Eph/ephrin family has also been involved in synaptogenesis and synaptic plasticity (reviewed in Ref. [83]). Regarding AMPARs specifically, Eph receptors have been involved in regulating their clathrin-mediated endocytosis, their localization, transsynaptic glutamatergic synaptogenesis, and downregulation during homeostatic plasticity [84–87]. Transsynaptic binding of presynaptic ephrinBs to postsynaptic EphB receptors induces binding of GRIP1 to EphB that is necessary for the induction of mossy fiber LTP [88]. Postsynaptic ephrinB3 interaction with GRIP1 was also shown to specifically promote the formation of shaft synapses in hippocampal cultures. The same study showed that ephrinB3 knockout mice display reduced shaft synapses, while spine density was unaffected [89]. Interestingly, synaptic AMPAR expression is also reduced in ephrinB3 knockout mice, and ephrinB3 regulates the anchoring and stability at the synapse of another crucial synaptic scaffolding protein, PSD95 [90, 91]. GRIP1–EphB2 interactions are also important for dendritic development. GRIP1 transports EphB2 to the dendritic compartment via its binding to KIF5 and by doing so regulates dendritogenesis [24]. EphrinB ligands recruit GRIP1 and GRIP2 to lipid rafts upon activation by EphB2, suggesting a role of GRIP1 proteins as scaffolds for large multiprotein complexes downstream of Eph/ephrin signaling [21]. As AMPAR internalization rate is increased in raft-depleted neurons, this supports a role of such GRIP-scaffolded complex in AMPAR stabilization at the surface [92]. In fact, activation of ephrinB2 reverse signaling was shown to regulate the insertion and stabilization of AMPARs at the surface and ephrinB2 knockout neurons displayed an enhanced constitutive AMPAR internalization and impairment in synaptic transmission [93]. A serine residue (Ser-9) in ephrinB ligands was found to regulate the binding to GRIP1 (Fig. 1). In a parallel study, the PDZ-binding site of ephrinB2 was shown to be required for both LTP and LTD at the CA3–CA1 hippocampal synapse [94]. Recently, additional insight on the regulation of AMPA receptor insertion at the synaptic membrane came from studies that described a cooperation model between the ephrinB2 ligands and the Reelin receptor apolipoprotein E receptor 2 (ApoER2 [95]). ApoER2 had been previously implicated in AMPAR trafficking following stimulation with Reelin and ApoER2 knockout mice showed an impaired LTP [96–98]. Mechanistically, this new study by Pfennig and colleagues shows that GRIP1 bridges a whole complex at the membrane that includes ephrinB2, AMPAR, and ApoER2. The formation of the complex is required for the new insertion and stabilization of AMPARs at the synaptic membrane, following either neuronal activity or stimulation with Reelin. Such complex is regulated by phosphorylation of the Ser-9 in ephrinB2 and is necessary for LTP maintenance [95].

### Protein interacting with C kinase (PICK1)

Originally identified as a partner of PKCα, PICK1 binds via its PDZ domain the C-terminus of the AMPAR subunits GluA2 and GluA3, like GRIP1 [20, 99]. PICK1 role in AMPAR trafficking has been extensively studied, and, in a relatively consensual model, acts as the counterpart to GRIP1 by regulating AMPAR endocytosis [20, 29, 31, 100, 101] (Fig. 1). PICK1 is, indeed, required for NMDAR-induced internalization of AMPARs [100, 102] as well as for LTD in the hippocampus [31, 103–105], the cerebellum [106], but also the cortex [107] and ventral tegmental area [108]. PICK1 is also able to bind activated PKCα and target it to the synapses, where it can phosphorylate GluA2-Ser880, thereby facilitating AMPAR endocytosis [101]. According to other studies, PICK1 regulates AMPAR recycling after NMDAR-induced internalization by retaining AMPARs intracellularly, but is not required for endocytosis itself, as PICK1 knockout neurons displayed an increased recycling rate of AMPARs to the surface but no impaired internalization [43, 109–111]. Both functions are not mutually exclusive, and actually not antagonistic, as both underlie the role of PICK1 in downregulating surface GluA2 expression. In addition, PICK1 has been involved in regulating calcium-permeable AMPAR trafficking, and its overexpression favors GluA2-lacking over GluA2-containing receptors [104, 112, 113].

PICK1 may also be involved in NMDAR-dependent LTP, as LTP is prevented by PICK1 knockdown and occluded by PICK1 overexpression [105]. However, another study found no defective LTP in PICK1 knockout slices [111]. The precise function of PICK1 may also be highly stimulus-dependent: while low NMDAR activation triggers an increase of PICK1 endosomal clustering (consistent with higher AMPAR endocytosis), a higher NMDAR activation (similar to LTP induction protocols) leads to the dissociation of GluA2–PICK1 complexes and an increase of surface GluA2 expression mediated by the *N*-ethylmaleimide-sensitive fusion protein (NSF), another AMPAR interactant, and calcium [110, 114]. Another study investigated LTP and LTD in juvenile and adult PICK1 knockout mice and found that both LTP and LTD were completely unaffected in juvenile mice, whereas a specific form of LTP only and multiple forms of LTD were impaired in adult mice; consistently, only adult mice displayed learning deficits [115]. These findings are strikingly similar to the phenotype shown by knockout mice for kidney and brain expressed protein (KIBRA), a gene related to human memory capabilities; KIBRA binds PICK1 and forms a complex with AMPARs, and its deletion impairs hippocampal LTP and LTD, and thereby learning, in adult mice, but not juvenile [116]. The precise function of PICK1 appears, therefore, highly contextual, depending on the brain region, age, and stimulus. Finally, in addition to LTD and LTP, PICK1 is also involved in homeostatic scaling up, which is occluded in PICK1 knockout neurons; it is not, however, required for scaling down [117].

Like GRIP1, PICK1 function in AMPAR trafficking is regulated by posttranslational modifications. While Thr82 phosphorylation on PICK1 prevents GluA2 binding, Ser416 phosphorylation by glycogen synthase kinase (GSK) 3β is required for PICK1 interaction with GluA2 [118, 119]. PICK1 palmitoylation by DHHC8 is required for cerebellar LTD [120]. Interestingly, the monoubiquitination of PICK1 by parkin1 triggers the excessive potentiation of the acid-sensing ion channels (ASICs), which also bind PICK1 PDZ domain; channel hyperactivity can lead to excitotoxicity, thereby potentially linking PICK1 and the neuronal degeneration seen in Parkinson’s disease [121].

In addition to GluA2/3, PICK1 binds a multitude of other proteins, some of which also modulate the regulation of AMPAR trafficking by PICK1. For instance, upon calcium release from the ER, PICK1 forms a complex with calcium/calmodulin-dependent protein kinase type II (CaMKII) and GluA2 that facilitates GluA2 exit from the ER and trafficking towards the dendrites [122] (Fig. 7). PICK1 can also act directly as a calcium sensor, and PICK1-GluA2 interactions are increased upon calcium stimulation. Thus, PICK1 binding to calcium is required for NMDAR-induced AMPAR internalization or their intracellular retention [102, 111]. Calcium also regulates the formation of a complex including PICK1, NSF, GluA2, and the SNARE protein β soluble NSF attachment protein (βSNAP), which modulates surface GluA2 expression, as NSF stabilizes AMPARs at the surface by destabilizing GluA2–PICK1 complexes [123, 124].

In addition, PICK1 also regulates spine morphology by binding to the small GTPase Arf1, F-actin, and the actin-nucleating complex actin-related protein Arp2/3 (Fig. 1). The formation of this complex, indeed, inhibits actin elongation and branching promoted by Arp2/3, which underlie vesicle trafficking and spine morphogenesis. Consistently, Arf1 binding, by preventing PICK1-Arp2/3 interaction, impairs NMDAR-induced AMPAR endocytosis and spine shrinkage during LTD [125–127]. Interestingly, PICK1 also binds the Rho family members Cdc42 and Rac1, providing a direct mechanistic link from AMPAR stimulation to the regulation of spine morphology [128]. One study, however, reported that PICK1 did not bind Arp2/3 nor F-actin directly, but was involved in nondirectional organelle motility driven by myosin motors [129]. PICK1 was also involved in axonal trafficking via its newly identified partner the kinesin-binding protein syntabulin [130], which might, therefore, act as a motor protein for PICK1 regulation of presynaptic AMPAR trafficking [131, 132]. Recently, PICK1 was also shown to interact with a central player in clathrin-mediated endocytosis, the adaptor protein AP2, which bridges cargo proteins and clathrin. This interaction is required for AMPAR clustering and internalization upon NDMAR stimulation [133].

Another important binding partner of PICK1 is islet cell autoantigen 69 kDa (ICA69), which blocks synaptic targeting of PICK1 and, concomitantly, of AMPARs [134] and PKCα [135]. This role is not only important for plasticity, but also during development, as ICA69 negatively regulates synaptic trafficking and clustering of GluA2-containing AMPARs by PICK1 during synaptogenesis, an important step for synapse maturation [136]. Furthermore, PICK1 bridges the protein kinase C and casein kinase substrate in neurons (PACSIN) family members PACSIN1 and 2 in a complex with AMPARs; this interaction depends on the phosphorylation of the PACSIN proteins and is required for AMPAR endocytosis and cerebellar LTD, but also regulates the subsequent AMPAR recycling to the surface [137, 138]. The vesicle sorting protein sortilin-related VPS10 domain-containing receptor 3 (SorCS3) also binds PICK1, and the impaired PICK1 localization and subsequent AMPAR synaptic targeting in SorCS3 knockout mice are proposed to underlie their deficits in cerebellar LTD and hippocampal learning behaviors [139, 140]. Finally, PICK1 also interacts with tetraspanin 7 (TSPAN7), in which mutations have been found in some types of X-linked intellectual disability. By competing with GluA2 to bind PICK1 PDZ domain, TSPAN7 limits PICK1 availability to AMPARs and, therefore, stabilizes them at the surface. PICK1/TSPAN7 interaction also regulates spine morphogenesis [141].

In addition to its PDZ domain, PICK1 possesses a bin–amphiphysin–Rvs (BAR) domain, through which it binds lipids; this binding regulates synaptic targeting of PICK1, and, therefore, of AMPARs, and consequently, mutating the BAR domain decreases synaptic surface clustering of AMPARs and LTD [103, 106]. Additional studies have focused on the reciprocal influences of the BAR and PDZ domains, as well as the N- and C-terminal domains of PICK1, and their importance for AMPAR trafficking [47, 142]. The BAR domain has also been proposed to enable PICK1 to form long-distance interactions and thereby facilitate its scaffolding of multiple membrane-bound proteins [143]. Contradictory findings were reported regarding PICK1 higher structure ([129]; see also Refs. [144, 145]). Further studies are required to delineate PICK1 structure and its functional influence on AMPAR trafficking.

### Membrane-associated guanylate kinase (MAGUK) family

MAGUKs form a large family of PDZ domain-containing scaffolding proteins that are essential for the development and plasticity of synapses. The MAGUK family is divided into several subclasses based on their domain organization, including the Discs large homologs (DLGs), calcium/calmodulin-dependent serine protein kinase (CASK), and palmitoylated membrane proteins (MPPs) [146].

The DLG family has been associated with AMPAR trafficking and plasticity for years. However, until recently, a direct association with AMPARs had only been demonstrated for synapse-associated protein 97 (SAP97 or DLG-1), which binds the C-terminus of the GluA1 subunit. Other DLG members such as PSD93 (DLG-2), PSD95 (DLG-4, or SAP90), and SAP102 (DLG-3) were found to bind directly NMDARs, but not AMPARs [147–149]. Intriguingly, PSD95, SAP97, SAP102, and the relatively unknown MPP2, were recently shown to belong to AMPAR native macromolecular complexes [10]. More detailed studies are now required to dissect this newly discovered association between DLG family members and AMPARs.

#### Postsynaptic density (PSD) 95 and 93

The prototypical member of the DLG family is PSD95, which was nearly simultaneously identified as a postsynaptic protein in rat synaptosomes and as SAP90 in the presynaptic compartment of Purkinje cells, prefiguring the wide array of functions served by the protein at both sides of the synaptic cleft [150, 151]. PSD95 appears to play a pivotal role in plasticity, and mice impaired for PSD95 signaling display, indeed, learning deficits and abnormal anxiety-like behavior [152, 153]. As a scaffold protein, PSD95 affects spine density and morphology. For example, spine number and size are increased upon overexpression, while PSD95 knockdown prevents spine morphological development, but also spine growth and stabilization after LTP induction [154, 155]. In addition, PSD95 drives the maturation of glutamatergic synapses, i.e., the insertion of AMPARs into synapses that previously only contained NMDARs, which converts them from “silent” to “functional” synapses [154]. Many studies modulated the levels (overexpression or knockdown) of PSD95 to investigate the function of PSD95 in synaptic plasticity, and the scenario is thought extremely puzzling. On one hand, several studies have shown that PSD95 overexpression increases the proportion of AMPAR expressing synapses, AMPAR synaptic clustering, and AMPAR-mediated synaptic transmission and, as a consequence, occludes LTP, since the strengthened synapses cannot further potentiate [7, 154, 156–159]. Very intriguingly, PSDS95 overexpression enhances LTD [156, 157]. On the other hand, the converse strategy (reducing PSD95 levels or using a ligand-binding deficient mutant) produces even more confusing results. In the adult hippocampus, LTP is, indeed, intact [155] or even (somewhat surprisingly) enhanced [152, 160, 161]. However, in the developing superior colliculus [162] and in the barrel cortex [158], LTP is blocked. On the other hand, in mice with lower PSD95 levels or functionally impaired PSD95, LTD is disrupted in the adult hippocampus [152, 155, 161], but it is intact in the developing hippocampus [153] and superior colliculus [162].

There are likely several mechanisms behind all this diversity of phenotypes. First, PSD95 functions appear clearly developmentally and spatially regulated. As the conversion of silent to functional synapses primarily takes place in the developing brain, it is not surprising to see a differential effect of PSD95 between the young and adult brain. In addition, PSD95 is not the only synaptic MAGUK; PSD93 and SAP102 are also present and may undertake similar functions, which might explain why PSD95 is not unconditionally required for LTP and why not all synapses are equally affected in PSD95 knockout mice ([159] and see below). Finally, PSD95 acts at multiple levels at the synapse—on the stability of the PSD itself, and also as a scaffold for a wide array of proteins in addition to AMPARs and NMDARs, all of which may be affected upon the changes of PSD95 levels. PSD95 may, therefore, also act not as a primary regulator of plasticity, but as a mediator. Regarding LTD, its major involvement seems to transduce NMDAR-mediated calcium influx by scaffolding additional proteins required for LTD-induced AMPAR endocytosis [163].

PSD95 does not only play a role in Hebbian plasticity; it has also been involved in homeostatic synaptic scaling, in an age-dependent fashion. Indeed, scaling up is only affected by PSD95 knockdown in young cortical neurons, while scaling down is impaired by PSD95 knockdown or overexpression, in young or adult cortical neurons [205]. This differential involvement of PSD95 is likely mediated by different domains and interaction partners of PSD95, some of which have been shown to be also required for scaling down [205].

**Fig. 2:**
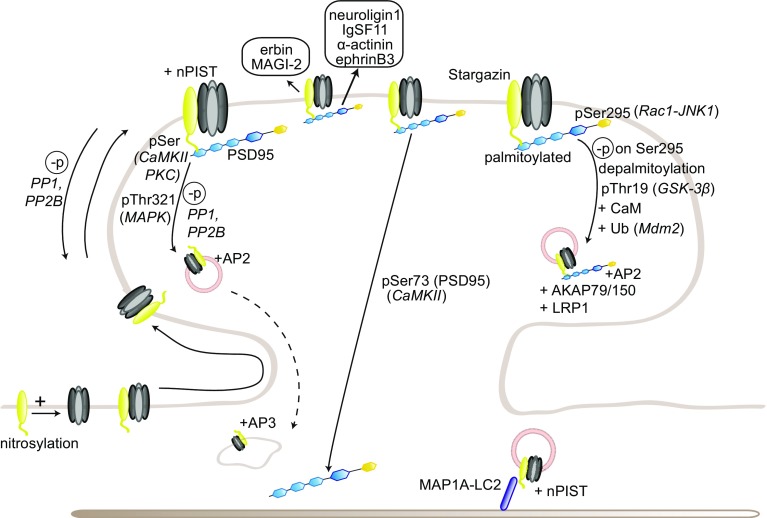
Regulation of AMPAR trafficking by PSD95 and TARP γ2/stargazin. PSD95 and TARP γ2/stargazin regulate AMPAR synaptic trapping. PSD95 anchors stargazin-bound freely diffusing AMPARs at the PSD, thereby enhancing synaptic strength. Stargazin phosphorylation in its cytoplasmic tail promotes PSD95 binding; upon dephosphorylation, stargazin-bound AMPARs are endocytosed or diffuse out of the synapse. Additional posttranslational modifications on PSD95 or stargazin modulate this binding in either direction. Stargazin also regulates AMPAR synaptic trafficking by interacting with MAP1A-LC2

The role of PSD95 as a scaffold protein for AMPARs has been extensively studied in the last decade. Mechanistically, PSD95 stabilizes AMPARs at the synaptic surface by scaffolding nanodomains at the synapse which are preferentially enriched in AMPARs rather than NMDARs. Such concentration in nanodomains, which enhances synaptic efficacy and is activity-regulated [164–166], involves the formation of a complex with the transmembrane AMPAR regulatory protein (TARP) γ2 (stargazin) necessary for the correct targeting and diffusion of AMPARs to the synaptic surface [7, 167–169] (Fig. 2). PSD95/stargazin complexes have also been suggested to regulate AMPAR “slots” at the synapse, where freely diffusing AMPARs are “trapped” to regulate synaptic strength; the precise molecular mechanisms underlying the formation of these slots are, however, still unclear (reviewed in Ref. [170]). In addition to stargazin, PSD95 also binds other TARPs, including γ3 and γ8, but the function of this interaction in synaptic plasticity has not been further investigated [171]. Surface AMPAR diffusion toward synapses is required for hippocampal LTP and for in vivo contextual learning [172]. Nanodomain formation also involves the capture of diffusing AMPARs by the adhesion molecules neurexin/neuroligin, which assemble them in PSD95 scaffolds in competition with the preexisting synapses [173]. Moreover, PSD95 has been recently shown to interact with the adhesion molecules immunoglobulin superfamily member 11 (IgSF11) and α-actinin, both of which are involved in AMPAR stabilization at the synapse [174, 175]. IgSF11 knockdown leads to a decreased AMPAR clustering and increased surface motility, suggesting that IgSF11 might also be involved in regulating the formation or maintenance of the nanodomains [174]. α-Actinin knockdown phenocopied PSD95 knockdown and knockout, as there is a reduction in the number of synapses but not in AMPAR content [175].

Two isoforms of PSD95 have been identified, which differ at their N-terminus. The α isoform is predominant and normally palmitoylated; under baseline conditions, it masks the effects of the β isoform. The regulation of synaptic strength by either isoform is activity-dependent [176, 177]. Palmitoylation, mediated by DHHC-containing palmitoyl transferases and a/b-hydrolase domain-containing 17 (ABHD17) depalmitoylase, is crucial for PSD95 sorting, synaptic targeting, its integration into the PSD lattice, and the clustering of AMPARs in nanodomains [178–183]. Palmitoylation also regulates PSD95 binding to stargazin and is prevented by an interaction with calmodulin (CaM). CaM binding enables the calcium-induced release of PSD95 and its anchored proteins from the postsynaptic membrane, a necessary step for the reorganization of the PSD following NMDAR-induced LTD [183, 184]. Consistently, palmitoylation is induced by chronic activity blockade during homeostatic scaling up, leading to an increased synaptic AMPAR clustering. CaM binding to PSD95 was very recently shown to mediate AMPAR internalization during synaptic scaling down [185, 186]. In addition to CaM binding, CaMKII itself also positively regulates PSD95 trafficking out of the spines following LTP induction by phosphorylating PSD95 on Ser73, which blocks LTP and LTP-induced spine growth, suggesting that CaMKII modulates the end of LTP-triggered cascades [183, 187]. Other phosphorylation sites of PSD95 include Ser295, whose phosphorylation by the Rac1/c-Jun-N-terminal pathway 1 (JNK1) pathway enhances synaptic targeting of PSD95 and AMPAR clustering and whose dephosphorylation is required for AMPAR internalization during LTD [188]; and Thr19, whose phosphorylation by GSK-3β is *a contrario* required for AMPAR endocytosis and LTD [189]. Recently, Ser561 phosphorylation by partitioning-defective (Par) 1 kinases was shown to function as a structural switch between open and closed conformations of PSD95; a phosphodeficient mutant favors an open conformation, increasing PSD95 synaptic stability and its ability to act as a scaffold by binding multiple proteins [190]. Ser561 phosphorylation is also required for AMPAR internalization following NMDAR activation. In addition, multiple phosphorylation sites modulating protein interactions have been newly identified in PSD95, but their role in synaptic plasticity remains to be investigated [191]. Finally, PSD95 is also ubiquitinated by the E3 ligase mouse double minute 2 (Mdm2) upon NMDAR activation, leading to its removal from the surface; this process is regulated by the kinase Cdk5 and modulates PSD95 binding to the clathrin adaptor protein complex AP-2, providing a mechanistic link between NMDAR activation and AMPAR endocytosis. And, indeed, blocking this ubiquitination prevents subsequent AMPAR endocytosis [192, 193].

In addition to the N-terminal PDZ domains, PSD95 contains also a C-terminal Src homology 3 (SH3) and guanylate kinase (GK) like domain. PSD95 interaction with A kinase anchoring protein (AKAP) 79/150 via its C-terminal SH3 and GK domains is necessary for NMDA-induced AMPAR endocytosis [194, 195]. PSD95 may also mediate AMPAR internalization via its complex with GluA1 and low-density lipoprotein receptor-related protein 1 (LRP1), a protein involved in regulating synaptic integrity; LRP1 knockdown, indeed, leads to accelerated turnover and decreased surface GluA1 and GluA1-induced neurite growth [196]. Finally, PSD95 binds also directly ephrinB3, which stabilizes it at the synapse; activity-induced phosphorylation of ephrinB3 Ser332 by mitogen activated protein kinase (MAPK) prevents this association and leads to an increased PSD95 turnover [91].

A last factor to be taken into consideration when studying MAGUK proteins is, as mentioned before, the presence of multiple family members at the synapse—presence which may itself differ depending on the age, brain region, and neuronal type considered. For instance, the closely related PSD93 is present at the postsynapse and displays a similar but not identical expression pattern to PSD95 in the adult rat brain, with a unique pattern in the cerebellar Purkinje cells [197]. Like PSD95, several isoforms of PSD93 have been identified—6 in total—with different impacts on AMPAR- and NMDAR-mediated functions; the most abundant isoforms in the hippocampus can also be palmitoylated [176, 198]. PSD93 can be phosphorylated by the kinases Fyn and extracellular signal-regulated kinase (ERK); it also mediates NMDAR phosphorylation by Fyn, an important regulatory modification of NMDAR function [199–201].

PSD93 and PSD95 seem to be present in a largely non-overlapping subset of synapses, and may, therefore, confer different properties to the synapses for which they are main MAGUK ([159, 202, 203], but see [204]). This differential pattern may explain why not all synapses are similarly affected in PSD95 knockout mice [159, 160]. Contrary to PSD95, PSD93 knockout mice display normal LTD, but an impaired LTP [161]. PSD93 is also able to support on its own scaling down in young cortical neurons, but not scaling up, nor scaling down or up in adult neurons [205]. In addition, in Purkinje cells at least, PSD93 binds the microtubule protein MAP1A, suggesting a potential role in regulating the dendritic trafficking of its partners, such as NMDARs and PSD95, with which it can form heteromers [197, 206].

#### Synapse-associated protein (SAP) 102

SAP102, originally identified as the first synaptic protein binding NMDARs, regulates NMDAR synaptic clustering and clearance and mediates NMDA-dependent plasticity and spine remodeling. Consistently, SAP102 knockout mice display impairments in ERK-dependent LTP and spike-time dependent plasticity, as well as cognitive deficits [207–211]. SAP102 also regulates AMPAR clustering at immature synapses. SAP102 knockdown strikingly leads to the removal of all AMPARs from the surface during synaptogenesis [159]. Synapse maturation requires the switch from SAP102 to PSD95 and PSD93, and SAP102 only supports AMPAR trafficking in mature synapses to compensate for the simultaneous knockdown of the normally predominant PSD95 and PSD93 [212]. SAP102 also functionally interacts with PSD95 (potentially by direct binding or via TARPs) and regulates its ability to enhance AMPAR-mediated synaptic transmission [213]. Contrary to PSD95 and PSD93 localization and general immobility in the sole PSDs, SAP102 is localized in the whole spine volume, in and around PSDs, and is highly mobile, a feature depending on actin and glutamate receptor activation [204, 214, 215]. This suggests that SAP102 function might be more related to AMPAR trafficking towards the synapse rather than stabilization at the surface. Recently, SAP102 synaptic targeting and spine motility were found to be regulated via phosphorylation by casein kinase II (CKII) of its Ser632 residue, a residue present in one of the three isoforms of SAP102. Phosphorylation of Ser632 was found to be induced by activity, leading to an increase of SAP102 density and stability at the synapse [216].

SAP102 interacts as well with neurobeachin, a brain-specific AKAP that regulates AMPAR trafficking [217–219]. SAP102 also forms a complex with EphB2 and the Ras guanine nucleotide exchange factor (RasGEF) effector Kalirin7 and regulates surface EphB2. Mechanistically, SAP102 is necessary for actin reorganization, synapse formation, and AMPAR synaptic trafficking upon EphB activation by ephrinB [220].

Further insights about the role of PSD95 and SAP102 come from studies where all three MAGUK proteins involved in regulating baseline transmission, i.e., PSD95, PSD93, and SAP102, were knocked down simultaneously by RNAi in organotypic slices [221, 222]. This knockdown causes a vast reduction in global AMPAR and NMDAR transmission, due to a decrease in glutamate receptor containing synapses; the synaptic strength of surviving synapses is, however, unaffected [221]. MAGUK loss triggers first a reduction in quantal postsynaptic currents, which then recovers via a consolidation process involving calcium channels, CaMKII and GluA2, similarly to a homeostatic process [221]. Furthermore, the knockdown leads to a decreased PSD size, a large loss of the PSD95 containing vertical filaments structuring PSDs and concomitantly of AMPAR or NMDAR complexes, and a subsequent decrease in synaptic transmission [222]. Collectively, these results strongly suggest a basic role for MAGUK proteins as a PSD scaffold, on which glutamate receptor trafficking is then grafted. These roles seem restricted during synaptogenesis, as MAGUK knockdown in adult CA1 in rats only slightly affected synaptic transmission acutely, causing a significant reduction only after several weeks; *a contrario*, the same procedure in young rats immediately leads to a large reduction continuing until adulthood [223]. Interestingly, MAGUK loss in the adult dentate gyrus leads to a similar effect as in a young CA1, emphasizing the role of MAGUK proteins during development. Consistently, a very recent study investigating SAP102 expression in adult and aging hippocampi suggests a developmentally and subregionally regulated role, which can be altered during neurodegenerative diseases such as Alzheimer’s [224].

#### Synapse-associated protein (SAP) 97

**Fig. 3:**
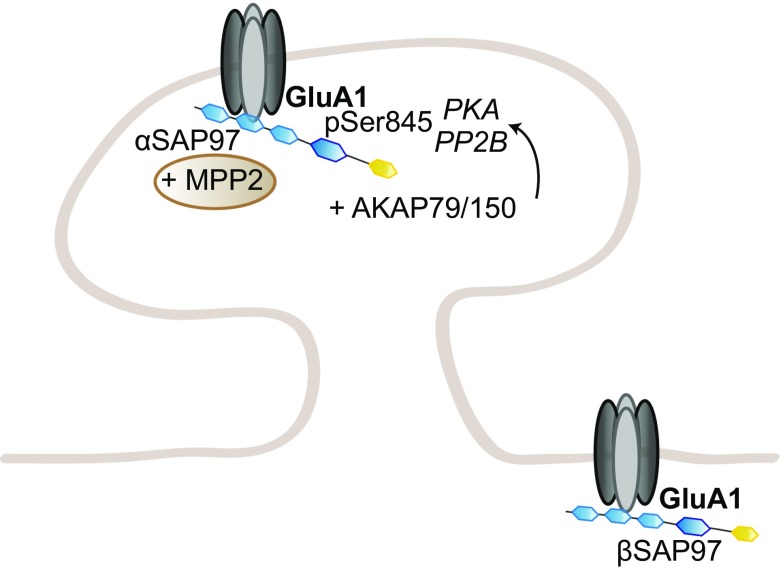
Regulation of GluA1 trafficking by SAP97. SAP97 specifically regulates GluA1-containing AMPARs in an isoform-specific manner. βSAP97 directs GluA1 to the extrasynaptic pool, while αSAP97 preferentially targets GluA1 to synapses

SAP97 occupies a special place in the MAGUK-DLG family as it specifically binds GluA1, and not GluA2–4; this specificity depends on a small sequence outside of GluA1 canonical PDZ-binding sequence [147, 149]. SAP97 appears to be involved in GluA1 early trafficking to the dendritic membrane, as well as the regulation of the extrasynaptic and synaptic pools of AMPAR at the surface [225–228] (Fig. 3).

Like PSD95, the N-terminus of SAP97 exists in two isoforms, the palmitoylable α isoform and the non-palmitoylable, L27 domain-containing β isoform. While the α isoform regulates AMPAR-mediated transmission in an activity-independent manner, the β isoform, most abundant, does it in an activity-dependent and CaMKII-regulated manner [176]. αSAP97 is directly targeted to the PSD, while βSAP97 is present at the perisynaptic regions [227]. These results are consistent with earlier reports of βSAP97 expression at the edges of PSDs in synapses expressing homomeric GluA1 channels or heteromeric GluA1-containing AMPARs; this pattern, in opposition to PSD95 and PSD93, points towards a role of βSAP97 in recycling GluA1 receptors in and out of the PSD [204, 226, 229]. Structurally, the presence of the L27 domain enables SAP97 to adopt an open conformation, indicating that αSAP97 and βSAP97 have a compact or extended conformation, respectively, providing a structural basis for the functional differences observed between the isoforms [230]. In addition, βSAP97 L27 domain is crucial for SAP97 dimerization and the modulation of synaptic strength; overexpression of L27-mutated SAP97 potentiates GluA1-dependent transmission, but does not affect synaptic delivery, suggesting a role for βSAP97 dimers in regulating GluA1 expression at the surface [231]. βSAP97 also contains an I3 splice variant in the hook region between the SH3 and GK domains, which includes a binding site for the actin-binding protein 4.1 N, another AMPAR interactant; overexpression of βSAP97-I3 drives an increase in spine head size, as well as in surface AMPAR expression and synaptic transmission [226].

Intriguingly, further investigation of the specific roles of SAP97 isoforms reveals a common effect on synaptic plasticity via distinct mechanisms; indeed, while overexpression of both isoforms impairs LTP and enhances LTD, αSAP97 acts solely on synaptic AMPARs, while βSAP97 regulates extrasynaptic AMPA and NMDARs [228]. αSAP97 overexpression increases the synaptic pool of AMPARs, thereby occluding further potentiation by LTP; βSAP97 overexpression directs AMPARs and NDMARs at the perisynapse, thereby preventing LTP induction. Conversely, βSAP97 knockdown increases the synaptic pool of AMPA and NMDARs. Consistently, overexpression of both isoforms was recently reported to increase AMPAR pool at the surface, but by different mechanisms [232]. αSAP97 acts, indeed, at the PSD itself, increasing its size and, therefore, the number of AMPAR slots, but also at the presynaptic clusters; βSAP97, on the other hand, increases AMPAR density at the PSD edges and its immediate surrounding regions, but not within the PSD. Taken collectively, these studies emphasize a different role of αSAP97 and βSAP97 in regulating the synaptic and perisynaptic pools of GluA1-containing AMPARs, respectively.

In addition to a role at the synapse, several studies suggest a function of SAP97 in early trafficking to the synapse. SAP97 association with GluA1 was reported to occur mainly in the early secretory pathway, at the ER or Golgi apparatus, with only a small portion of GluA1 receptors bound to SAP97 at the synapse [225]. Consistently, SAP97 expression was found mainly in regions coinciding with ER and Golgi apparatus; its SH3 and GK domains were not required for its role in GluA1 delivery [233]. Moreover, SAP97 binds directly myosin VI and SAP97–GluA1–myosin VI complexes are detected in vesicles; disrupting SAP97–myosinVI interaction leads to a decrease in synapse number, surface AMPAR, and synaptic strength, emphasizing a role for SAP97 as adaptor protein in GluA1 transport [234, 235].

SAP97 also acts as a scaffold protein bridging GluA1 to other partners. One of them is AKAP79/150, which itself interacts with PKA and protein phosphatase 2 regulatory subunit (PP2B); SAP97, thus, provides a platform regulating GluA1 phosphorylation at Ser845, and thereby GluA1-mediated synaptic transmission [236]. AKAP79/150 binds a specific isoform of SAP97 containing the C-terminal I3 or I5 sequence; at this position, SAP97 can also be phosphorylated on Ser39 by CaMKII, a step necessary for synaptic delivery of SAP97, and concomitantly GluA1; this phosphorylation prevents AKAP79/150–SAP97 binding [237, 238]. This interaction is also important for the switch from GluA1-containing to GluA4-containing AMPARs between the early and late stages of behavioral conditioning; SAP97–AKAP79/PKA–GluA1 complexes are formed initially, and then replaced by SAP97-kinase suppressor of Ras (KSR) 1/PKC–GluA4 complexes, and at both times, SAP97 interacts with PSD95 to deliver the corresponding AMPAR subunit at the synapse [239]. This suggests a more general role for SAP97 in AMPAR trafficking, even without a direct interaction to GluA2–4, and is consistent with a previous study showing an involvement of SAP97–PSD95 interaction in regulating GluA1 trafficking at the synapse [240]. SAP97 can also compensate when PSD95 and PSD93 are knocked down, and using conditional SAP97 knockout mice, SAP97 involvement in GluA1 trafficking was shown to be most important during early development [241]. SAP97 also binds to the MAGUK CASK; this interaction stabilizes SAP97 in its extended conformation, which preferentially colocalizes with NMDARs, contrary to the unbound SAP97, which adopts the compact conformation and colocalizes with GluA1 receptors [242]. Consistently, CASK–SAP97 complex mediates NMDAR sorting through a specific secretory pathway, separated from AMPARs [243].

In addition, SAP97 links GluA1 to a disintegrin and metalloproteinase 10 (ADAM10), a membrane-bound secretase cleaving neuronal amyloid precursor protein (APP) at the synapse. SAP97 ability to bridge the ADAM10–GluA1 complex was significantly decreased in the hippocampi of Alzheimer’s patients, and ADAM10 and GluA1 synaptic levels were consequently lower; in rodents, such a decrease in ADAM10 levels favors the amyloidogenic pathway [244, 245]. In addition to Alzheimer’s disease, altered SAP97–GluA1 interactions have also been involved in schizophrenia [246]. Finally, SAP97 also has a transsynaptic action; SAP97 overexpression at the postsynapse increases presynaptic size and function, via the recruitment of GluA1, but also adhesion molecules, including cadherins, integrins, and the EphB/ephrinB family at the membrane [247]. This transsynaptic effect is exerted primarily by αSAP97, although βSAP97 can also slightly increase presynaptic Bassoon levels [232]. This indicates an extensive role of SAP97 in regulating GluA1 delivery to the dendrites and relative abundance in the perisynaptic and synaptic pools, but also in synapse formation and function.

#### MAGUK p55 subfamily member (MPP) 2

Barely anything is known about the neuronal functions of MPP2. MPP2 is localized at the postsynapse and interacts with many PSD proteins, including itself, PSD95, SAP97, and actin, but also CaMKIIα, suggesting a role as a scaffold protein [248]. Recently, MPP2 was found to interact also with guanylate kinase-associated protein (GKAP) and the synaptic cell adhesion molecule (SynCAM1) 1, a protein also involved in structuring PSDs [249]. MPP2 also interacts with small conductance-activated potassium SK2 channels and positions them properly at the synapse, a crucial element for their role in synaptic plasticity [250]. A similar role for AMPARs is open for investigation.

## Transmembrane AMPAR regulatory proteins (TARPs)

Transmembrane AMPAR regulatory proteins form the first family to have been identified as auxiliary proteins to neuronal AMPARs, and more generally to any neurotransmitter-gated ion receptor [148, 251, 252]. Structurally, TARPs are part of the calcium channel γ subunit (CACNG) family, which comprises of eight members, CACNG 1–8 or γ1–γ8. CACNG1 and 6 function primarily as calcium channel auxiliary proteins in skeletal muscles and do not interact with AMPARs at all [253]. The TARP family includes the other six members, the prototypical γ2 or stargazin, γ3, γ4, γ5, γ7, and γ8, and is subdivided into two types based on sequence homology and function: Type I contains γ2, γ3, γ4, and γ8, and is itself split into Type Ia (γ2 and γ3) and Type Ib (γ4 and γ8), while Type II encompasses the remaining γ5 and γ7 [252]. The spatial and temporal expression pattern of TARPs is partially overlapping and, therefore, single TARP knockout mice do not show any gross phenotype, with the exception of γ2, suggesting partial functional redundancy [252, 254, 255]. Differences have also been observed in the stoichiometry of the TARP–AMPAR complexes, thereby influencing AMPAR function; a complex includes up to four TARP/AMPAR for γ2 and γ3, but seldom more than two TARP/AMPAR for γ4. The stoichiometry varies also according to the neuronal cell type [256–258]. Type I TARPs can bind all GluA subunits, but in a mutually exclusive fashion—one TARP type per complex [259]. Finally, TARPs do not only regulate AMPAR trafficking, but also its physiological properties such as agonist/antagonist response, gating, and kinetics [252]. We will, in this review, focus on the function in AMPAR trafficking of the TARPs identified by Schwenk and colleagues as auxiliary proteins to native AMPARs, i.e., γ2, γ3, γ4, γ7, and γ8 [10].

### TARP γ2/stargazin

Stargazin was first identified as a disrupted gene in the mutated chromosome 15 of the stargazer mice, a spontaneous mouse mutant displaying absence epilepsy [260]. Subsequent studies in the stargazer cerebellum revealed that stargazin interacts with AMPARs and PSD proteins, including PSD95, PSD93, SAP97, and SAP102 [167]. Since then, stargazin was found to regulate several aspects of AMPAR trafficking, including synaptic targeting [167, 261, 262], surface receptor delivery [263], stabilization [264], diffusion [265, 266], and endocytosis [267], and is, therefore, involved in plasticity processes such as LTP and LTD [268, 269] and synaptic scaling [270, 271]. Stargazin function is highly specific for AMPARs, as surface delivery of NMDA and kainate receptors is stargazin-independent [272].

The multiple functions of stargazin are preferentially mediated by its different domains, with the C-terminal cytoplasmic tail and the extracellular domain influencing mainly receptor trafficking and channel properties, respectively [273–275]. Stargazin binds GluA subunits intra- and extracellularly, and its interaction with the glutamate-binding domain of AMPARs is important for channel desensitization [276]. On the other hand, the C-terminal domain contains a membrane sorting signal important for stargazin regulation of AMPAR synaptic delivery [261]. The first reconstruction of stargazin structure, performed in a lipid bilayer, showed that the C-terminus strongly interacts with the lipidic membrane, leading to an extended open conformation and thereby facilitating protein interactions [277]. Several recent studies further investigated the AMPAR–stargazin complex [278–282]. They dissected the role of stargazin in modulating AMPAR gating, first via the destabilization of closed receptors by its initial binding, and by the subsequent stabilization of AMPAR activated state by electrostatic interactions between stargazin and the receptors [278, 281, 282]. Its positioning below the AMPAR ligand-binding domain enables stargazin to modulate receptor gating by regulating intra- and inter-dimer interactions, but also to act as a scaffold bridging the receptors to other regulatory proteins, emphasizing the intricate connections between the various functions assumed by stargazin [279, 280].

Stargazin plays an essential role in AMPAR synaptic targeting; surface expression of the receptors is, indeed, reduced in stargazer cerebella, where stargazin is the major TARP [167]. As mentioned before, stargazin–PSD95 interaction is essential for its regulation of AMPAR synaptic targeting [167] (Fig. 2). In the ER, stargazin has been suggested to act as a chaperone-like protein ensuring the correct folding of AMPAR subunits, although it differs from classical chaperones in its continuous association with its targets outside of the ER [263]. AMPARs in the stargazer cerebellum are not completely N-glycosylated and, therefore, do not properly mature [259] and the unfolded protein response (UPR) is upregulated in the cerebellum of stargazer mice [263]. However, another study found that stargazin played a major role at later stages of AMPAR maturation, and that its chaperone-like duties may become necessary only upon high levels of AMPAR synthesis [283]. Stargazin forms, indeed, a complex with GluA2 and MAP1A light chain (MAP1A-LC2), which is important for AMPAR early trafficking, likely prior to entering the PSD, as the complex does not include PSD proteins [284]. At a later stage of trafficking, stargazin also bridges AMPARs and PSD95 to the Golgi-enriched neuronal isoform of protein interacting specifically with TC-10 (nPIST), an important interaction for surface expression and clustering of AMPARs [285].

Stargazin–PSD95 interaction is also crucial for controlling synaptic abundance of AMPARs by regulating their diffusion between extrasynaptic and synaptic pools [7, 168]. Consistently, enhancing PSD95 binding by extending the length of stargazin cytoplasmic tail was more recently shown to increase AMPAR trapping at synaptic sites [286]. Phosphorylation of Thr321 within stargazin PDZ-binding domain by PKA disrupts its binding to PSD95 and thereby synaptic clustering and function of AMPARs [287, 288]. Intriguingly, Thr321 can also be phosphorylated by MAPK, but with different consequences: blocking PKA-mediated phosphorylation prevents LTP, while preventing MAPK-mediated phosphorylation blocks LTD, but not LTP [268]. These results are consistent with MAPK role in AMPAR removal during LTD; they also suggest that PSD95 is required for some, but not all, of stargazin functions. Taken collectively, these studies underlie the role of phosphorylation as bidirectional switch for stargazin role in AMPAR trafficking and in plasticity.

Stargazin can also be phosphorylated at nine serine residues of its cytoplasmic tail; the phosphorylation is carried out by CaMKII and PKC, and dephosphorylation by phosphatase 1 (PP1) downstream of PP2B/calcineurin [289]. Phosphorylation on these residues increases stargazin binding to PSD95, but prevents its binding to lipid bilayers; these mutually exclusive interactions add a further level of complexity to stargazin regulation of AMPAR synaptic delivery [290]. Interestingly, synaptic NMDAR activity can trigger serine phosphorylation and dephosphorylation, depending on the stimulation protocol and the type of plasticity induced—phosphorylation for LTP; dephosphorylation for LTD [289, 291]. Consistently, calcineurin-mediated dephosphorylation of stargazin is also required for cerebellar LTD [269]. Mechanistically, dephosphorylated stargazin binds the clathrin complex AP-2, triggering clathrin-mediated endocytosis of AMPARs, which are then delivered to late endosomes/lysosomes upon binding of stargazin to AP-3 [267]. Interestingly, the role of stargazin in endocytosis might also be stimulus-dependent: glutamate-induced desensitization indeed leads to the dissociation of stargazin/AMPAR complexes, and while stargazin remains stably at the membrane, AMPARs are subsequently internalized [264]. Here, stargazin passively sets up AMPARs for internalization simply by unbinding them, contrary to the active part taken in NMDAR-induced endocytosis. Another study indicates that, following the decreased affinity between stargazin and AMPARs due to glutamate-induced desensitization, AMPARs leave the synapse by lateral diffusion rather than endocytosis; this increased surface motility facilitates their replacement with naive receptors [266]. These results are not mutually exclusive; several conformations of desensitized AMPARs, with potentially different affinities for stargazin and, therefore, set up for separate pathways, have also been reported [292].

In addition to AMPAR synaptic delivery and cycling, stargazin phosphorylation by CaMKII regulates their lateral diffusion: NMDAR-induced calcium influx activates CaMKII, leading to stargazin phosphorylation and increasing its binding to PSD95, thereby trapping PSD95-stargazin-AMPAR complexes at the synapse [265]. Similarly, stargazin serine phosphorylation is required for the new synaptic insertion of AMPARs during tetrodotoxin (TTX)-induced synaptic upscaling; it is also important for the experience-dependent development of the retinogeniculate synapse, and it is increased upon visual deprivation during that phase [270]. Conversely, inducing synaptic downscaling leads to the dephosphorylation of stargazin serine residues, an increased surface motility of stargazin and AMPARs, and a higher AMPAR endocytosis, the latter being mediated by stargazin interaction with AP-2 and AP-3 [271].

Interestingly, stargazin is also nitrosylated by nitric oxide (NO) under basal conditions, enhancing its binding to AMPARs and their surface expression; nitrosylation is increased upon NMDAR activation, adding a further regulatory residue to NMDAR-governed stargazin modulation of AMPAR trafficking [293]. Stargazin can also be glycosylated, although the influence of this posttranslational modification on stargazin functions remains to be investigated [294].

In addition to DLG-MAGUK and the clathrin adaptor complex, stargazin also binds several other proteins regulating its functions in AMPAR trafficking. Stargazin interacts, for instance, with the adaptor protein Erbin in cortical parvalbumin-positive (PV) interneurons; this interaction is essential for stargazin stability at the surface, and, therefore, AMPAR surface expression and function [295]. Consistently, stargazer mice were shown recently to display altered AMPAR subunit composition in PV interneurons, leading to a loss of the feedforward inhibitory circuit in the somatosensory cortex; these data provide a mechanistic link between stargazin and the absent epileptic seizures characteristic of the stargazer mouse models [296, 297]. This may stem from a differential regulation of AMPAR subunits trafficking by stargazin; indeed, while stargazin targets GluA2 subunits to the dendritic surface, it directs GluA1 receptors only to the dendrites, but not the surface [298]. Stargazin exerts additionally a protective effect for GluA1-containing receptors against lysosomal degradation, but not for GluA2/GluA3 heterodimers. Interestingly, stargazin also binds another MAGUK protein, membrane-associated guanylate kinase 2 (MAGI-2), a scaffold protein interacting with many proteins involved in neuronal morphology, such as dendrin, axin, and catenins; this interaction is important for MAGI-2 localisation at the synapse and provides a link between AMPARs, TARPs, and neuronal morphology [299]. Consistently, Type I TARPs influence the development of cortical pyramidal dendritic trees, stargazin specifically at later stages [300]. Taken collectively, these studies underlie the importance of stargazin as a scaffolding protein, bridging AMPAR trafficking and other crucial neuronal processes.

### TARP γ3, γ4, and γ8

Functionally, γ3, γ4, and γ8 were classified as TARPs when it was shown that their transfection in stargazer cerebellar granule cells could restore AMPAR-mediated responses [264]. Like stargazin, they are enriched in the PSD and are important for surface expression of AMPARs in the brain regions where they are expressed; γ3 is mainly, but not exclusively, present in the cerebral cortex, γ4 in the olfactory bulbs, striatum, and glia, and γ8 in the hippocampus [264]. Contrarily to stargazin, γ3 and γ8, γ4 expression reaches its peak during embryonic and early postnatal development and slowly decreases afterwards [264]. Like stargazin, γ3 and γ8 interact with PSD95 and PSD93; it is currently unknown, but probable, for γ4 [171].

γ3 and γ4 knockout mice do not show any gross phenotype; neither do γ3/γ4 double knockout mice [254, 255, 301]. However, stargazer (*stg*)/γ3 double knockout mice die a couple of weeks after birth, a stronger phenotype than pure stargazer mice [254]. This suggests that the lack of phenotype in γ3 knockout mice comes from a compensation by overlapping TARPs, i.e., stargazin, but also γ8. Indeed, synaptic AMPAR content and function is unaffected in *stg*/γ3 double knockout hippocampal neurons, where γ8 is still expressed, but impaired in cerebellar Golgi neurons, where γ8 is not. This loss of synaptic AMPARs reflects specifically a decrease in GluA2-containing receptors, implying a subunit-specific trafficking regulation by stargazin and γ3. Interestingly, Golgi neurons are unaffected in single knockout mice, suggesting that stargazin or γ3 alone is sufficient to regulate AMPAR trafficking and function [254].

**Fig. 4:**
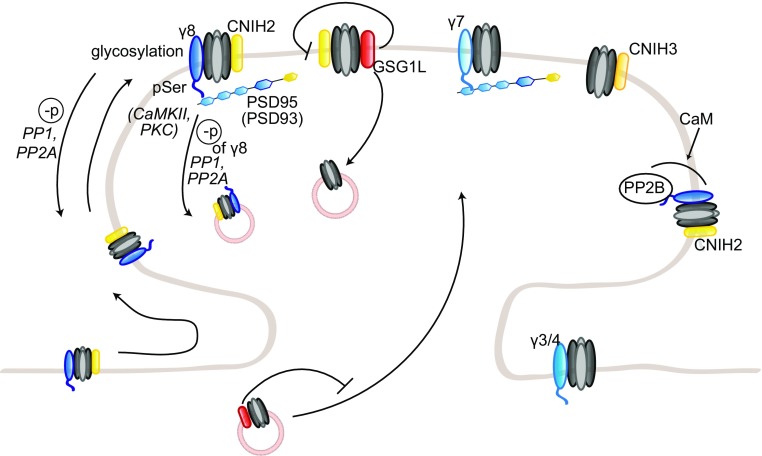
Regulation of AMPAR trafficking by TARP γ3, γ4, γ7, and γ8, CNIH and GSG1L. TARP γ3, γ4, γ7, and γ8, CNIH2 and 3, and GSG1L modulate AMPAR trafficking to and at the synapse. γ3, γ4, and γ8 and CNIH2 together, target AMPARs to the extrasynaptic pool. γ8 and CNIH2 also cooperatively regulate AMPAR lateral diffusion to and anchoring at the synapse, by binding MAGUK proteins. TARP γ7 and CNIH3 are also present at the synapse. GSG1L, on the other hand, promotes AMPAR endocytosis and negatively regulates AMPAR trafficking to the synapse

The phenotype of *stg*/γ4 double knockout mice appears to be background-dependent, as this strain has been reported viable by Menuz and colleagues, but with very low levels of birth by Letts and colleagues [255, 301]. To compensate for the lack of viable *stg*/γ4 double knockout mice in their hands, Letts and colleagues crossed γ4 knockout mice to milder alleles of the stargazin gene, waggler and stargazer3J. These double mutants were viable and displayed an increased number of seizures compared to single waggler or stargazer3J mice; this worsened phenotype indicates that γ4 acts as a seizure repressor, although the exact mechanisms are unclear [301]. This may be related to the specific modulation of AMPAR function by γ4; indeed, while most properties are regulated to a similar extent to stargazin, AMPAR desensitization is much more strongly modulated by γ4 [302]. Triple knockout mice for stargazin, γ3 and γ4 die at birth from apnea and are paralyzed, pointing towards a developmental role for γ3 as well [255]. Intriguingly, AMPAR targeting to the perisynaptic and synaptic surface is unaffected in cortical and spinal neurons [255]. This suggests a compensation by another TARP; as it is expressed in both neuronal populations shortly after birth and can influence AMPAR function at that age, γ8 is a likely candidate [255]. In the nucleus accumbens, γ4 was found to mainly localize in perisynaptic membranes, contrary to the synaptic localization of stargazin, suggesting a preferential role in regulating the perisynaptic AMPAR pool [303] (Fig. 4).

While γ8 knockout mice appear normal, they do show a strong impairment of AMPAR trafficking, contrary to γ3, γ4, or γ7 knockout mice [8]. γ8 loss leads to a striking 85% reduction of GluA proteins in the hippocampus, where γ8 is primarily expressed; the remaining receptors are mislocalized and do not reach the dendritic membrane. Consistently, γ8 overexpression enhances surface AMPAR expression, indicating a role in AMPAR synaptic delivery. In γ8 knockout mice, both extrasynaptic and synaptic AMPAR pools are affected; however, the extrasynaptic pool is much more depleted, suggesting a selective delivery of the available AMPARs to the synaptic pool. Basal synaptic transmission and LTP are impaired, likely due to an insufficient extrasynaptic pool of AMPARs; on the other hand, LTD is intact, implicating that γ8 is not involved in NMDAR-induced AMPAR endocytosis [8, 304]. The stronger reduction in surface AMPARs in *stg*/γ8 double knockout mice suggests that stargazin mediates the trafficking of the few AMPARs expressed at the membrane in γ8 knockout mice [8]. γ3 may also complement γ8 and stargazin, but as *stg*/γ3/γ8 mice die embryonically, it is difficult to assess. On the other hand, γ4 is most likely not involved, as γ3/γ4/γ8 triple knockout mice, who are viable and fertile, do not show a stronger phenotype than γ8 knockout mice [255].

Another mouse line lacking γ8 on a different background shows a similar phenotype, as surface AMPARs are reduced in hippocampal synapses, while intracellular AMPARs are unaffected [305]. However, the synaptic and extrasynaptic pools are here similarly reduced; as the global abundance of AMPARs is also much less decreased (55% instead of 85% reduction), this is still consistent with a preferential delivery to the synaptic pool, with a greater amount of AMPARs now available to the extrasynaptic pool [305]. Both studies indicate a role for γ8 in AMPAR delivery to the surface and the activity-induced mobilization of the extrasynaptic pool by lateral diffusion (Fig. 4). Consistently, γ8 was recently described as the only TARP expressed at the nonperforated synapses of the hippocampal Schaffer collaterals onto CA1 pyramidal cells, which have a lower AMPAR density compared to perforated synapses, where stargazin and γ3 were also detected [306]. In *stg* perforated synapses, compensatory γ8 upregulation failed to completely rescue surface AMPAR expression; conversely, in γ8 knockout perforated synapses, compensation was successfully carried by stargazin upregulation. This suggests that stargazin and γ8 regulate AMPAR trafficking and synaptic density at different levels—stargazin being required for high synaptic expression and γ8 for low density, basal expression; the lack of changes in γ3 knockout mice points toward a different role for γ3 in the hippocampus [306].

While the PDZ-binding domains are identical between Type I TARPs, γ8 possesses unique stretches at the C-terminus and diverges from stargazin at the other two intracellular domains, suggesting the existence of a different regulatory mechanism [289]. Indeed, swapping all cytoplasmic domains between stargazin and γ8 is enough to completely exchange the synaptic AMPAR phenotype between the two TARPs, showing that intracellular domains as a whole regulate synaptic AMPAR trafficking [307]. The role of the PDZ-binding domain is still ambiguous; it has been hypothesized to be required for the synaptic localization of AMPAR/TARP complexes via PSD95 binding by TARP, and, therefore, subsequent LTP. Consistently, a recent study reported that LTP was impaired in single-cell genetic experiments, where GluA1 homomers tethered to PDZ-binding domain-lacking γ8 were expressed on a triple GluA knockout background [308]. However, knockin mice expressing γ8 lacking its PDZ-binding domain display an impaired basal transmission, but normal LTP, suggesting that different mechanisms are regulating synaptic AMPAR localization in these two events [304]. Another possibility is compensation by other TARPs in the knockin mice, or a differential regulation for AMPAR heteromers compared to GluA1 homomers; additional work is required to elucidate this model. In both studies, however, the decreased basal transmission is attributed to lower synaptic AMPAR content, while the extrasynaptic pool is unaffected, suggesting that the backbone of γ8 is important for extrasynaptic AMPAR targeting, in line with other studies [8, 304, 306]. Consistently, and contrary to stargazin PSD localization, γ8 is preferentially found in the extrasynaptic membranes [304, 309].

Like stargazin, γ8 is subjected to several posttranslational modifications. γ8 is notably phosphorylated at several C-terminal serine residues by CaMKII and PKC, and dephosphorylated by PP1 and/or PP2A, but not PP2B [289, 309]. γ8 phosphorylation was recently found to be required for LTP, as demonstrated by an impaired LTP in mice expressing phosphodeficient γ8; this is consistent with the requirement for LTP of the γ8 backbone, outside of the PDZ-binding domain [310]. Contrary to stargazin, which does not bind PP2B, but is a substrate, γ8 binds the phosphatase PP2B/calcineurin in a CaM-dependent manner, but is not a target; via this association, γ8 may regulate AMPAR phosphorylation levels and, therefore, their trafficking during basal conditions, but also LTP and LTD [311]. γ8 is also N-glycosylated, a modification essential for its trafficking to the membrane and surface expression; moreover, unglycosylated γ8 on γ8 knockdown background is unable to rescue the decreased AMPAR surface expression, suggesting that the maturation of γ8 is required for the correct synaptic delivery and surface expression of AMPARs [294].

γ3, γ4, and γ8 have, therefore, all been involved in regulating AMPAR trafficking. γ8 is now well established as the major TARP in the hippocampus, and its main role appears to modulate AMPAR delivery to the extrasynaptic pool and its lateral diffusion to the synapse. On the other hand, the exact roles of γ3 and γ4 are still uncertain due to the general compensation by the preponderant stargazin and γ8, and require further work.

### Tarp γ7

TARP γ7 was first identified as a regulatory protein for voltage-gated calcium channels; its low-sequence homology to Type I TARPs and its different structure suggested that it did not function as a TARP, like γ5 [312]. However, γ7 was then shown to be highly expressed in the cerebellum, especially in Purkinje cells, granule cells, and stellate cells [313, 314]. γ7 is enriched in the PSD and binds all GluA subunits and PSD95 (Fig. 4); furthermore, γ7 modulates AMPAR trafficking and gating, and its overexpression enhances glutamate-evoked AMPAR currents [313]. γ7 regulates also specifically GluA2-containing AMPARs and its modulation depends on GluA2 editing [315].

In addition, γ7 and stargazin are abundantly detected at various cerebellar asymmetrical synapses (such as the parallel or climbing fiber synapses onto Purkinje cells) but absent from symmetrical synapses (basket or stellate cell synapses onto Purkinje cells) [314]. Knockout mice for stargazin (*stg*), γ7, or double knockout for *stg*/γ7 showed a selective reduction in synaptic AMPAR subunits content—GluA2/3 were markedly affected in *stg*, GluA1/4 moderately in γ7 knockout, and GluA2/3/4 in double knockout mice. Intriguingly, these losses were predominant at different asymmetrical synapses, pointing towards a cooperation between stargazin and γ7 to promote AMPAR synaptic expression [314]. The lack of phenotype displayed by γ7 knockout mice and the cerebellar impairments shown by the stargazer mice suggest, however, that γ7 function is relatively small and unable to fully compensate for the lack of stargazin. Indeed, as shown by the lack of further impairment in *stg*/γ7 double knockout compared to *stg* mice, γ7 contribution to excitatory transmission is negligible [316]. Nonetheless, in the double knockout mice, the climbing fiber-to-Purkinje cell synapses are affected and their excitatory transmission impaired, suggesting a specific modulation by γ7 of this class of synapses. Consistently, selective deletion of stargazin in Purkinje cells on a wild-type background did not cause any motor defects, while this deletion on a γ7 knockout background severely impaired multiple motor behaviors [316]. These results suggest that stargazin and γ7 cooperation is specifically regulating the climbing fiber input onto Purkinje cells, and that this collaboration is essential for motor control.

On the other hand, γ7 has also been implicated in regulating the trafficking and perisynaptic accumulation of GluA2-lacking, calcium-permeable AMPARs in stellate and granule cells [317]. These receptors are, however, unable to cluster at the synapse and AMPAR-mediated transmission is then ensured by “TARPless” AMPARs, suggesting that neither stargazin nor γ7 is required for surface expression and a role for γ7 in trapping AMPARs in the extrasynaptic pool [317]. Consistently, γ7 selectively increases calcium-permeable AMPAR expression at the synapse of granule cells and downregulates GluA2-containing, calcium-impermeable AMPARs [318]. Conversely, however, another study has shown the requirement of stargazin for surface AMPAR expression in cerebellar stellate cells [262]. All in all, the role of γ7 in cerebellar granule and stellate cells appears still controversial; its role in Purkinje cells seems so far unchallenged. Its small contribution to AMPAR trafficking may indicate a greater role in unrelated functions; indeed, γ7 regulates the stability of specific mRNAs via its interaction with the RNA-binding heterogeneous nuclear ribonucleoprotein (hnRNP) A2 and promotes neurite outgrowth via the retrograde transport of signaling vesicles [319, 320].

## Cornichon proteins (CNIHs)

CNIH2 and 3 are transmembrane proteins first identified as cargo transporters in *Drosophila*, and only more recently as AMPAR auxiliary subunits in a proteomic analysis of AMPAR complexes [321, 322]. CNIHs appear to bind the majority of AMPARs in the rat brain, even more so than TARPs. Like TARPs, CNIHs are involved in both AMPAR trafficking and channel kinetics, as CNIH overexpression increases surface AMPAR expression and slows down inactivation and desensitization, suggesting a role in stabilizing the channel in an open, active configuration [322]. CNIH2 and 3 expression patterns in the brain show a peak during the first two postnatal weeks and a slow decrease over time, contrary to GluA subunits; however, the proportion of CNIH bound to AMPARs remains constant, indicating a specific reduction in AMPAR-free CNIH and suggesting a switch from general cargo transporter to AMPAR auxiliary subunit [323].

Immediately after CNIH identification as auxiliary subunits, two partially conflicting studies regarding their exact functions were published [324, 325]. While Shi and colleagues found a major role in cargo trafficking, with very little surface expression in hippocampal neurons [325], Kato et al. showed that CNIH2 is not only present in postsynaptic densities, but it also associates with TARP γ8 and AMPARs in a complex cooperatively regulating channel gating and pharmacology, specifically AMPAR desensitization [324]. Intriguingly, CNIH2 levels were strikingly reduced in γ8 knockout mice, similarly to the reduced levels of GluA1/2 subunits observed in these mice [8, 324]. CNIH proteins lack a synaptic targeting PDZ-binding site, which is consistent with a role for AMPAR trafficking towards the extrasynaptic pools together with γ8. In agreement, Shi et al. showed that CNIH2 overexpression is able to partially rescue extrasynaptic, but not synaptic AMPAR pool in stargazer granule cells; coexpression with γ8, as found by Kato and colleagues, enhances that rescue [324, 325]. Furthermore, the roles of CNIH2 as cargo transporter and auxiliary subunit are not mutually exclusive; in hippocampal neurons, CNIH2 mediates primarily AMPAR transport between the ER and Golgi complexes, but interaction with GluA subunits leads to its escape from the early anterograde pathway and its transport with the receptor to the cell surface, where it stays associated and becomes a membrane protein [326] (Figs. 4, 7). A similar double role in trafficking and gating regulation was found for the sole CNIH homolog in *C. elegans*, suggesting a conserved dual function [327].

The role of CNIH proteins may also depend on the neuronal cell type, especially via the differential expressions of TARP isoforms. While CNIH2 differentially modulates AMPAR gating by regulating the stoichiometry of TARPs within AMPAR complexes, it only reaches the surface when coexpressed with γ8, explaining the localization and channel gating differences between γ8-expressing hippocampal neurons and γ8-lacking Purkinje cells [328, 329] (Fig. 4). In addition, the role of CNIH proteins may also be GluA subunit-dependent. CNIH2 and 3 bind preferentially to GluA1 subunits and facilitate the transit to and expression at the surface of GluA1/A2 heteromers over GluA2/A3 receptors; CNIH2 and 3 conditional knockout mice show a deep reduction in AMPAR function due to a retention of immature receptors at the ER, a shift in AMPAR transmission consistent with a loss of GluA1 receptors, and impaired LTP [330]. In agreement, both CNIH2 and 3 are associated at the surface with GluA2-lacking, calcium-permeable AMPARs and regulate their desensitization [331]. However, another study reports that CNIH proteins bind equally to GluA1 and GluA2 subunits, and that the lack of binding to GluA2 might rise from a technical issue; in this report, CNIH2 was found to mediate the switch from fast to slow AMPAR-mediated transmission in hilar mossy cells, further emphasizing the role of CNIH in AMPAR kinetics [332].

Structurally, CNIH proteins bind AMPARs via their membrane-proximal domain; compared to CNIH1 and 4, CNIH2 and 3 have an additional extracellular loop interacting with the ligand-binding domain and transmembrane domain of GluA subunits and necessary to exert their regulatory functions [333, 334]. These elements are both bound by CNIH and γ8, but lead to a regulation of AMPAR gating in opposite directions by each auxiliary protein, providing a structural basis for the regulation by CNIH of γ8-mediated AMPAR de- and resensitization and the differential pharmacology induced by the binding of either auxiliary protein [334, 335]. Further studies are required to investigate the domains involved in regulating AMPAR trafficking from the ER to the membrane and to distinguish the specific roles of CNIH2 and 3.

## Germline-specific gene 1-like (GSG1L)

GSG1L was recently identified as an AMPAR auxiliary subunit with a structural similarity to the TARP family [10, 336]. In heterologous systems and *Xenopus* oocytes, GSG1L slows down AMPAR desensitization and deactivation, similarly to TARPs [10, 336]. In hippocampal CA1 neurons, however, GSG1L modulates AMPAR trafficking and gating in a very different way to TARPs, as it downregulates AMPAR trafficking to the synaptic and extrasynaptic pools and enhances deactivation and desensitization, thereby favoring a reduction in AMPAR function at the synapse [337] (Fig. 4). Furthermore, AMPAR endocytosis is promoted upon GSG1L overexpression and reduced in GSG1L knockout rats. LTP is enhanced in these rats, consistently with the increased availability of extrasynaptic AMPARs and the reduced endocytosis rate [337]. Intriguingly, GSG1L reverts CNIH2 modulation of AMPAR kinetics both in *Xenopus* oocytes and CA1 neurons; as loss of CNIH2 occludes GSG1L effects, GSG1L function might actually be exerted via CNIH2 [10, 337]. Remarkably, in hippocampal granule cells, while GSG1L also negatively regulates AMPAR trafficking and surface expression, the protein only influences AMPAR gating when overexpressed, but not upon knockout suggesting that, in this population, GSG1L is only required for synaptic trafficking and not for channel properties [338]. In the brainstem nucleus abducens, GSG1L acts as a chaperone protein during classical conditioning, which requires the consecutive delivery of GluA1-containing receptors, followed by GluA4-containing heteromers; the former are bound and chaperoned by γ8, and the latter by GSG1L, providing the necessary subunit specificity for the learning paradigm; these results further emphasize the role of GSG1L in AMPAR trafficking [339]. Structurally, the cytoplasmic region of GSG1L and especially the Loop1 domain are crucial to mediate its regulation of AMPAR function [337]. GSG1L binding to AMPARs induces a conformation promoting the desensitized state, providing a structural basis for its physiological functions [340, 341]. Further investigation is required to understand the specific role of GSG1L in conjunction with other auxiliary proteins.

## Shisa6 and 9 (cystine-knot AMPAR modulating proteins (CKAMP) 52 and 44)

**Fig. 5:**
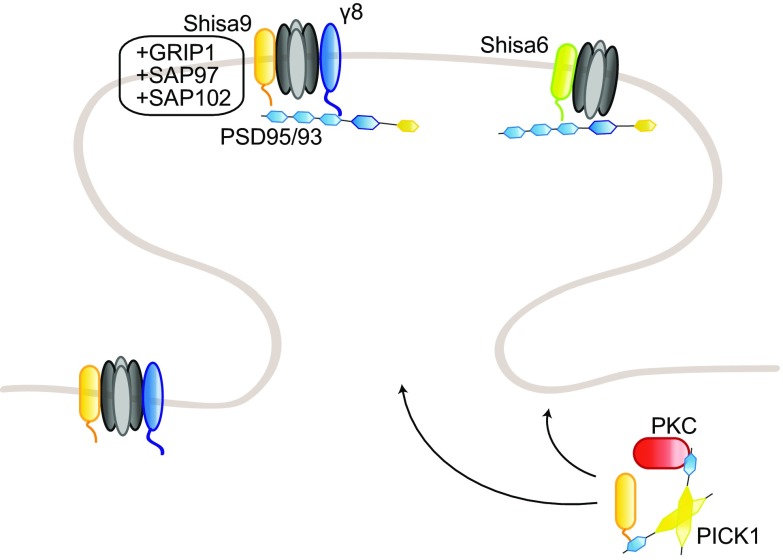
Regulation of AMPAR trafficking by Shisa proteins. Shisa6 and Shisa9 favor AMPAR stabilization at the synapse by binding to PSD95. Shisa9 is present in γ8-bound AMPAR complexes and binds several other PDZ domain-containing synaptic proteins; it also forms a complex with PICK1 and PKC upon trafficking to the synapse

The Shisa family of scaffold proteins are characterized by an N-terminal cysteine-rich and a proline-rich C-terminal regions and include several subclasses supporting a very wide range of cellular functions [342]; one subgroup is formed by the members Shisa6–9, which are also known after their function and molecular weight as cystine-knot AMPAR modulating proteins (CKAMPs) [343, 344]. Shisa9, or CKAMP44, was the first Shisa protein to be identified as interacting with AMPARs, but also with PSD95 and stargazin; Shisa9 is localized at the PSD and modulates AMPAR desensitization and short-term plasticity at hippocampal synapses [343] (Fig. 5). Shisa9 can bind AMPARs in complex with other auxiliary proteins, such as TARP γ2, γ4 or γ8, suggesting that they do not share the same binding site [345]. In the hippocampus, Shisa9 is lowly expressed in CA1 neurons and seems to play a very minor role; however, in the dentate gyrus, it is highly expressed and associated with AMPAR/γ8 complexes. Shisa9 binding to PSD95 and the reduction in AMPAR/γ8 complexes in Shisa9 knockout mice suggest that Shisa9 stabilizes AMPARs at the surface. Consistently, spine density is positively regulated by Shisa9 and γ8 [345]. Shisa9 knockout mice display a decreased surface AMPAR expression equally at the synaptic and extrasynaptic sites, indicating that Shisa9 does not regulate preferentially one of these pools. LTP in dentate granule cells was also unaffected in these mice, contrary to γ8 knockout mice, where it is strongly reduced, consistently with LTP at the Schaffer collaterals [8, 345]. AMPAR de- and resensitization and short-term plasticity is regulated by γ8 and Shisa9 in opposite directions; interestingly, this means that AMPAR desensitization in double knockout mice is actually similar to wild-type mice, as the knockout compensate each other [345]. Very recently, Shisa9 was shown to be crucial for the integration of visual inputs at the reticulogeniculate synapse, which depends on short-term depression of AMPARs; in agreement with the literature, Shisa9 attenuates AMPAR resensitization, thereby enhancing short-term depression and preventing neuronal hyperactivation, but it also promotes surface AMPAR expression and thereby synaptic strength, suggesting that Shisa9 exerts a carefully balanced modulation on the relay of visual signals [346].

Structurally, AMPAR binding requires a region downstream of Shisa9 transmembrane domain and the cystine-knot domain is essential for its modulation of AMPAR trafficking and gating [345]. In addition to PSD95, the cytosolic PDZ-binding domain of Shisa9 binds to several other scaffold proteins, including PSD93, SAP97, SAP102, GRIP1, and PICK1 [347, 348]. Disrupting PDZ interactions is enough to phenocopy the effect on AMPAR synaptic localization and transmission of Shisa9 knockout suggesting that at least one of these partners is essential for Shisa9 regulatory function [347]. Recently, interaction with PICK1 was shown to bridge Shisa9 with PKC, inducing Shisa9 phosphorylation; PICK1 association requires binding of another N-terminal domain of Shisa9, which is important for its regulation of AMPAR function, suggesting a possible competition between AMPAR and PICK1 [348]. As PICK1 interaction with PKC is important for its targeting to the membrane, this could provide a mechanism for the membrane targeting of Shisa9/PICK1, followed by a switch of Shisa9 to AMPAR/TARP/PSD95 complexes at the surface.

In addition to Shisa9, Shisa6, or CKAMP52, was also identified specifically as part of the native AMPAR complexes [10, 349]. Like Shisa9, Shisa6 is present at the PSD and binds PSD95 via its cytosolic domain, forming a complex with AMPARs and trapping them at the synapse; moreover, Shisa6 regulates AMPAR gating by preventing desensitization, thereby protecting hippocampal CA1 neurons from depression [349] (Fig. 5). Of note, while not part of the native complexes as described by Schwenk and colleagues [10], Shisa7 has also recently been found to associate with AMPARs and regulate their synaptic function; Shisa7 knockout mice impaired LTP initiation and maintenance, and decreased short-term and long-term contextual fear memory [350]. As the expression pattern of Shisa6–9 is developmentally regulated and as they differentially regulate AMPAR trafficking and gating in a cell-type-dependent manner [344], additional studies are necessary to determine more precisely their specific role in the various different brain regions.

## Leucine-rich repeat transmembrane protein (LRRTM) 4

LRRTM4 belongs to the four-member leucine-rich repeat transmembrane protein (LRRTM) family of cell adhesion molecules, and alone in this family has been reported as a member of native AMPAR complexes [10]. Little is known about LRRTM4 role in synaptic function. LRRTMs are predominantly expressed in the brain, with partially overlapping patterns; LRRTM4 itself is highly expressed from the end of embryonic development in several brain regions, including the cerebral cortex, the dentate granule cells and the CA3, but not CA1, pyramidal cells in the hippocampus, and a subset of cerebellar Purkinje cells [352]. The LRRTM family has been mainly characterized for its role as synaptic organizers (recently reviewed in Ref. [353]). LRRTMs are present at the postsynaptic sites; during development, they promote presynaptic differentiation in contacting axons [354], and as no effect on inhibitory synapses has been reported so far, their synaptogenic role appears specific to excitatory synapses [353]. Contrary to LRRTM1–3, which acts as receptors in *trans* for presynaptic neurexins, similarly to neuroligins, LRRTM4 interacts with glypicans (GPCs), a family of heparan sulfate proteoglycans [355–359]. More specifically, LRRTM4 binding to axonal GPC-4 induces their clustering on their respective side of the synaptic cleft, and inhibiting this interaction using heparinases blocks LRRTM4 synaptogenic function [357, 360]. GPC-4 additionally binds the LAR tyrosine phosphatase PTPσ, and the formation of the LRRTM4/GCP-4/PTPs complex mediates presynaptic differentiation and excitatory transmission [358]. In LRRTM4 knockout mice, dentate granule cells, but not CA1 pyramidal cells, exhibit a lower number of excitatory synapses; they also displayed lower synaptic levels of PSD95, an impaired excitatory (but not inhibitory) transmission and an inability to insert or stabilize new AMPARs at the surface following chemical LTP induction [360].

**Fig. 6:**
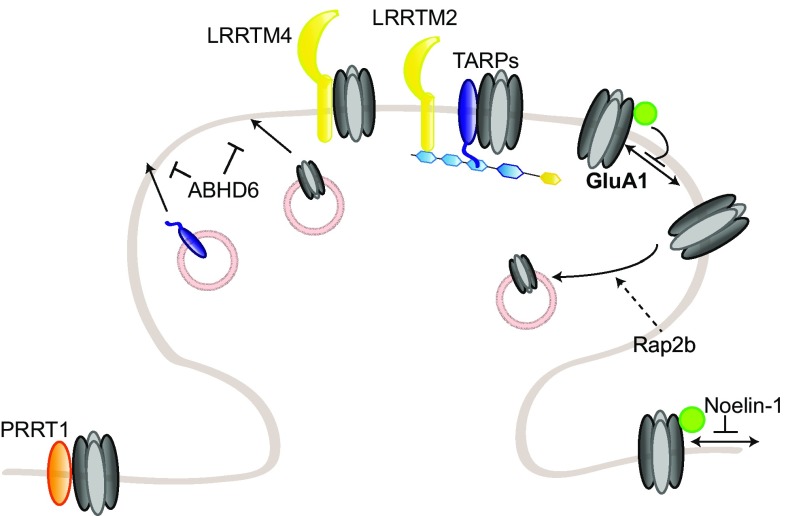
Newcomers in AMPAR complexes. Newly described constituents of synaptic AMPAR complexes include PRRT1, which targets AMPARs to the extrasynaptic pool; LRRTM proteins, which maintain the pool of surface AMPARs; ABHD6, which negatively regulates surface AMPARs and stargazin; Noelin-1, which negatively regulates lateral mobility of AMPARs (extrasynaptic GluA1- and GluA2-containing receptors; synaptic GluA1-containing receptors); and Rap2b, which has been shown to trigger AMPAR endocytosis indirectly, by recruiting effectors. A postsynaptic role of other interactants, such as neuritin, brorin, brorin-like or PRRT2, is still unknown

Interestingly, a regulatory role in AMPAR trafficking has also been found for LRRTM1 and 2. Indeed, LRRTM2 has been shown to directly bind PSD95 and regulate surface AMPAR trafficking [351]. Additionally, knocking down both proteins leads to decreased surface AMPARs following chemical LTP and an impaired LTP in the adult Schaffer collaterals [361]. These findings were very recently confirmed in genetic double knockout mice, where the complete deletion allowed Malenka and colleagues to uncover a role for LRRTM1 and 2 in basal AMPAR transmission, strongly suggesting a role in maintaining a sufficient pool of AMPARs at the surface [362]. Taken together, these results indicate a common role for LRRTMs in regulating AMPAR trafficking and function, albeit in different neuronal populations (Fig. 6). As LRRTM expression patterns are not completely mutually exclusive, further work is required to determine the existence of member-specific mechanisms.

## Proline-rich transmembrane (PRRT) proteins 1–2

PRRT 1 and 2 are type II transmembrane proteins; they were recently classified as members of the newly identified Dispanin family of transmembrane proteins, which are characterized by two transmembrane helices with several conserved motives [363]. The C-terminus of PRRT1 (or Dispanin subfamily D member 1, DSPD1) shares extensive similarity with the C-terminus of another Dispanin member, Dispanin subfamily C member 2 (DSPC2), initially identified as synapse differentiation induced gene (SynDIG) 1; hence the other name of PRRT1, SynDIG4 [364]. Interestingly, SynDIG1 is a known AMPAR interactant: it is associated with AMPARs at synaptic and extrasynaptic sites and promotes their clustering, and SynDIG1 distribution is regulated via activity-dependent palmitoylation [365, 366]. In vitro and in vivo, SynDIG1 also promotes the activity-dependent maturation of AMPAR-containing synapses by increasing synaptic AMPAR and PSD95 contents, thereby regulating synaptic number and strength ([365, 368]; but see [369]). PRRT1 presents a complementary expression pattern to SynDIG1 and is especially enriched in the CA1 region of the hippocampus; intriguingly, it is only present in the PSD in low amounts and mainly colocalizes with extrasynaptic AMPARs [364] (Fig. 6). Consistently, PRRT1 knockout mice mice show reduced extrasynaptic AMPARs, but also weaker synapses, impaired tetanus-induced (but not theta-burst induced) LTP, and cognitive impairments, suggesting that PRRT1 is required for specific forms of synaptic plasticity, possibly by ensuring the existence of a sufficient pool of extrasynaptic AMPARs [370]. On the other hand, PRRT2 (or Dispanin subfamily B member 3, DSPB3) has been identified as the causative gene for multiple paroxysmal disorders arising from different mutations (reviewed in Ref. [371]). PRRT2 is expressed in subpopulations within the cortex, hippocampus, cerebellum, and basal ganglia; it appears to be mainly a presynaptic protein and is only detected at low levels in the PSD [372]. At the presynapse, PRRT2 interacts with SNAP25, a component of the SNARE machinery, and synaptotagmins 1 and 2; it negatively regulates the priming of neurotransmitter-containing vesicles by blocking the SNARE machinery, thereby modulating synaptic transmission [372, 373]. In addition, PRRT2 also binds intersectin 1, a presynaptic scaffold protein [367]. Consistently with the observed symptoms, PRRT2 mutations found in human patients disrupt this regulation and thereby synaptic transmission [373, 374]. In addition, PRRT2 plays a role in neuronal migration and synaptic development [374]. At the postsynapse, barely anything is known: PRRT2 has been detected at the tip of dendritic spines and reported to restrict surface AMPAR levels [374, 375]. Additional studies are certainly needed to confirm and characterize its role as AMPAR auxiliary subunit.

## Neuritin

Neuritin is part of the neurotrophin family and was originally identified as candidate plasticity gene (cpg) 15 in a screen for plasticity-related genes in the rat hippocampus [376]. Neuritin has since then been established as a major player in activity-dependent remodeling of dendritic and axonal arborization by combining cell and non-cell autonomous actions: following the upregulation of its expression by activity, neuritin enhances axonal growth and branching, but can also be released from the axonal membrane by cleavage of its glycosylphosphatidylinositol (GPI) anchor, subsequently uptaken by the postsynapse, and thereby promoting dendritic arborization [377–382]. Neuritin thus supports the stabilization and maturation of active synapses and is thereby crucial for experience-driven neuronal network development, as shown by the disrupted visual receptor fields displayed by neuritin knockout mice [383]. In addition, neuritin knockout mice display delayed hippocampal development, decreased spine stability leading to a reduced spine density, and a lengthened learning process, although memory persistence is unaffected [384]. Neuritin also has been involved in synaptic transmission via its regulation of potassium and calcium channels [66, 385]. However, in addition to Schwenk and colleagues [10], neuritin has only been associated with AMPARs by a single study: Cantallops and colleagues showed that, in a non-cell autonomous manner, neuritin leads to the recruitment of AMPARs to the contacting postsynapses in *Xenopus* optic tectal neurons and the conversion of silent synapses to functional synapses [379]. These data are consistent with an association with AMPAR complexes; however, the mechanisms and additional possible functions during plasticity in mature networks are open to investigation.

## Noelin

**Fig. 7:**
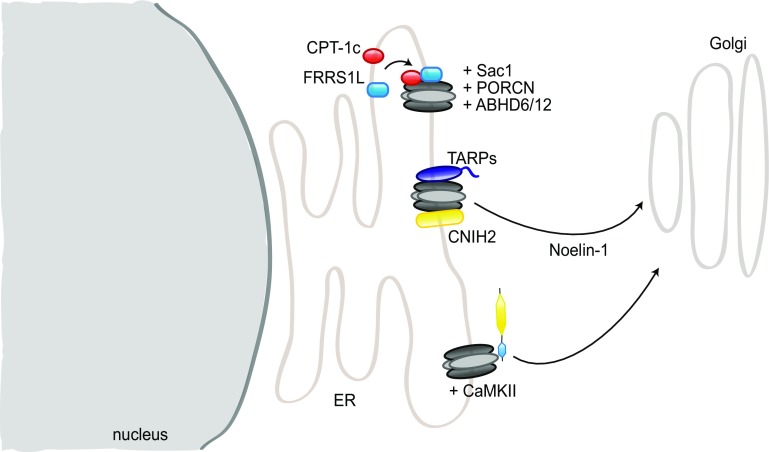
Endosomal constituents of AMPAR complexes. ER-specific AMPAR complexes ensure their proper biogenesis and prime them for synaptic partners, such as TARP and CNIH. These ER-specific interactants include CPT-1c and FRRS1L, which bind together, providing a platform for GluA subunits and subsequently Sac1, PORCN, and ABHD6/12 binding. Several TARPs, CNIH2, PICK1, and Noelin-1 have also been involved in regulating AMPAR early trafficking

Noelin is a secreted glycoprotein belonging to the larger olfactomedin family and transcribed in four alternative splice isoforms, Noelin 1–4, also known as olfactomedin 1–4 in zebrafish [386, 387]. Noelin isoforms play important roles during various stages of the development of the neural system and head structure [388–391]. Regarding plasticity, both Noelin-1 and 2 have also been shown by independent studies to interact directly with AMPARs [10, 392–395]. Noelin-1 interacts with additional AMPAR auxiliary subunits, such as TARP γ8 and neuritin; in addition, Noelin-1, 2, and 3 are able to form heterodimers and have a similar expression pattern, suggesting the possible coexistence of multiple Noelin isoforms within a given AMPAR complex [394, 395] (Fig. 6). Noelin-1 knockout mice display abnormal social- and anxiety-related behaviors and impaired olfaction, increased resting and activity-induced calcium concentrations in the hippocampus and olfactory bulbs, and upregulated calcium-dependent signaling pathways; however, AMPAR function was unaffected [392]. Consistently, Pandya and colleagues recently showed that Noelin-1 did not influence AMPAR channel properties [395]. On the contrary, Noelin-1 regulates the lateral mobility and interaction with the extracellular matrix of AMPARs, in a subunit-specific manner: increasing Noelin-1 amounts in hippocampal neurons affects both synaptic and extrasynaptic GluA1-containing AMPARs, but only extrasynaptic GluA2-containing AMPARs. This suggests that additional auxiliary subunits are involved in regulating the lateral mobility of synaptic GluA2-containing AMPARs [395]. In zebrafish double knockout for olfactomedin1 a/b, the homologs of Noelin-1, levels of synaptic AMPARs are significantly reduced; as GluA2 palmitoylation is decreased (but phosphorylation unaffected), this suggests that Noelin-1 regulates GluA2 maturation and thereby trafficking from the ER to the dendritic compartment, a role not mutually exclusive with regulating AMPAR lateral mobility [393] (Fig. 7). Another hypothesis would be that Noelin-1 enhances AMPAR surface stabilization, as it is present in lipid rafts, which have been shown to be important for this process [38]; however, the fact that AMPAR endocytosis is reduced in the mutant makes this possibility unlikely. Intriguingly, Noelin-1 is also present in the presynapse and interacts with SNARE proteins, which are also reduced in the presynaptic compartment in knockout fish [393]. Noelin-2 knockout mice show impaired behaviors and olfaction similar to Noelin-1 knockout mice, with additional slight motor defects; the composition of synaptic AMPAR complexes was also altered, with increased levels of GluA2, but decreased Noelin-1, PSD95, and CNIH-2 [394]. How this relates to AMPAR function, and which role plays Noelin-3, the additional isoform identified by Schwenk and colleagues, remain to be investigated.

Of note, another pair of secreted proteins, brorin and brorin-like, have also been identified by Schwenk and colleagues as AMPAR interactants [10]. Very little is known about them; they act as neural-specific bone morphogenetic protein (BMP) antagonists in mouse and zebrafish and as regulators of neural development [396–399]. Their expression in the adult brain leaves the door open for functions in postmitotic neurons, but extensive work is still required to identify an AMPAR-related role.

## Ras-related protein (Rap) 2b

Rap2b is one of the three alternatively spliced isoforms of Rap2, a member of the Ras family of small GTPases, which also includes Ras and Rap1. Only Rap2b has been identified as a member of native AMPAR complexes by Schwenk and colleagues [10]. This is rather surprising, as the sequence of these isoforms is over 90% identical, and no known Rap2 effector has shown so far isoform-selective activity in vitro [400]. However, they are differentially palmitoylated, and contrary to Rap2a and c, Rap2b does not require palmitoylation for its binding to the plasma membrane [401]. Multiple functions specific for Rap2b have already been characterized, many of which cancer-related (reviewed in Ref. [402]), but no study on Ras/Rap signaling in plasticity has so far focused on a given Rap2 isoform, and the available information, as presented in this section, is, therefore, applicable to all Rap2 isoforms. The Ras family regulates AMPAR trafficking bidirectionally: while Ras is important for AMPAR insertion and LTP, Rap1 and 2 mediate AMPAR removal during LTD and depotentiation, respectively [403–407]; reviewed in Ref. [408]) (Fig. 6). Rap2 activates JNK and induces AMPAR dephosphorylation, while Rap1 acts via p38 MAPK; neurons expressing a constitutively active Rap2 show reduced surface AMPAR levels and lower synaptic function, and are unable to maintain LTP [404, 409].

Together with Ras, Rap2 also mediates activity-induced synapse-specific AMPAR trafficking: Ras promotes transfer from the cytoplasmic to the synaptic pool, and Rap2 induces AMPAR removal from the synapse to the cytoplasm, enabling an activity-dependent reallocation of the receptors to other synapses [405] (Fig. 6). Consistently, Rap2 negatively regulates dendritic and axonal arborization: when constitutively active, Rap2 induces a decreased complexity and spine loss; intriguingly, despite a similar role in physiological plasticity, Rap1 does not affect neuronal morphology [409]. Transgenic mice expressing a constitutively active Rap2 show a similar phenotype, although Rap2 affects LTD but not depotentiation, and via ERK but not JNK signaling [410]. While these discrepancies are puzzling, this report is still consistent with a major role for Rap2 in AMPAR removal from the synaptic pool. Very recently, Zhang and colleagues studied the localization of Ras/Rap proteins in subcellular microdomains and showed that Ras mediates LTP via ER PI3K and lipid raft ERK, Rap1 transduces LTD via lysosomal p38 MAPK and Rap2 signals depotentiation via bulk membrane JNK, confirming and adding a further level of complexity to the current paradigm [407]. Efforts have so far focused on identifying coregulators and effectors of Rap2-mediated plasticity [406, 411], but not yet on the effects of its direct binding to AMPARs, which has yet to be confirmed independently. Further studies are thus required to tease out the specific role of Rap2b in AMPAR trafficking.

## a/b-hydrolase domain-containing (ABHD) 6 and 12

ABHD6 and 12 are monoacylglycerol lipases—a primary function a priori surprising in a review dedicated to AMPAR trafficking. However, they were both identified as constituents of the native AMPAR complexes and ABHD6 expression has been detected specifically in dendritic spines [10, 412]. ABHD6 overexpression leads to a decrease of AMPAR-mediated transmission and impaired LTP, while a knockdown has the opposite effect; NMDAR and GABA receptor-mediated transmissions are unaffected [413]. Moreover, surface AMPAR and stargazin are reduced in ABHD6 overexpressing cells, suggesting a role in restricting the surface pool of AMPARs (Fig. 6). In addition, this function does not require ABHD6 enzymatic activity, and is, therefore, also independent from the endocannabinoid pathway [413, 414]. Finally, ABHD6 binds GluA1–3 at their C-terminus and its effect is subunit-independent; it is also independent from stargazin [415]. Unexpectedly, ABHD6 and 12 were also identified as part of AMPAR complexes at the ER, suggesting a dual regulatory role, at least for ABHD6 (see below) [416].

## ER AMPARs interactants: carnityl palmitoyltransferase (CPT) 1C, ferric chelate reductase 1 like (FRRS1L), Sac1, and protein-serine *O*-palmitoleoyltransferase porcupine (PORCN)

CPT-1C is a brain-specific isoform of the depalmitoylating enzyme CPT1, mainly characterized for its role in food intake and energy homeostasis (reviewed in Ref. [417]). CPT-1C is expressed in hippocampal pyramidal cells and localized in the ER, in the soma but also in the spines; CPT-1C deficiency leads to a decreased number in mature spines and, consistently, impaired spatial learning [418]. Although this has been linked to CPT-1C enzymatic activity via ceramide depalmitoylation, this phenotype is reminiscent of the role of GluA2 in promoting spine density and maturation [419]. CPT-1C, indeed, interacts with AMPARs and enhances their surface level and function; however, consistently with earlier reports, it colocalizes with intracellular AMPARs, but not at the plasma membrane [420]. Furthermore, while GluA1 must be depalmitoylated at the C585 residue, CPT-1C is not the causative enzyme. CPT-1C knockout mice show reduced surface AMPAR amounts and function, due to a decreased translation efficiency; transcription and degradation are unaffected [421]. This reduced amount of synaptic AMPARs provides an additional mechanistic explanation for the decreased number of mature spines in CPT-1C knockout mice, and a mediation of CPT-1C action by AMPARs, beside ceramide, is a distinct possibility.

Recently, Brechet and colleagues reported that CPT-1C, together with FRRS1L and the phosphatidylinositide phosphatase Sac1, defines a distinct type of AMPAR complexes, located only at the ER, representing 15–20% of all AMPARs, and lacking all classical auxiliary subunits [416] (Fig. 7). CPT-1C and FRRS1L first bind each other, providing a platform for Sac1, and the ternary complex then associates with AMPARs in the ER. CPT-1C/FRRS1L knockdown leads to a decreased AMPAR function and number of functional AMPARs in the synaptic and extrasynaptic pools, strongly suggesting that, despite their exclusive location at the ER, these proteins do influence AMPAR trafficking, possibly through the priming of these early complexes for transfer to the Golgi apparatus and binding to other auxiliary subunits.

These results are consistent with a previous report on Sac1 involvement in AMPAR secretory trafficking; stress-induced palmitoylation of JNK3 led to its binding to and sequestration of Sac1, thereby reducing surface levels of AMPARs [422]. FRRS1L modulation of AMPAR function was also shown in two other recent studies, one of which detected FRRS1L in dynein vesicles, indicating a possible additional role in AMPAR trafficking beyond the ER [423, 424]. Notably, the impact of FRRS1L on synaptic function is independent of any enzymatic activity, as FRRS1L lacks a ferrochelatase domain, and is consistent with the symptoms of human patients suffering from FRRS1L loss of function, which leads to encephalopathies and severe intellectual disabilities; disease mutations do indeed disrupt FRRS1L binding to AMPARs, suggesting that these mutations impair the regulation of AMPAR early biogenesis by FRRS1L [416, 423].

Finally, another auxiliary subunit, PORCN, has been recently characterized as regulating AMPAR trafficking in the ER [414]. PORCN was first identified in *Drosophila* as an ER protein processing members of the Wnt pathway [425]. The porcupine family is highly conserved and several alternatively spliced isoforms exist, with different spatio-temporal expression patterns, but all encode transmembrane ER proteins [426]. While PORCN overexpression in hippocampal neurons has no effects, suggesting saturating protein levels, its knockdown leads to a dramatic reduction in total amounts of AMPARs and several auxiliary subunits, including TARP γ2, γ8, and CNIH2. Consistently, in conditional PORCN knockout mice, synaptic and extrasynaptic surface levels of AMPARs are decreased, as well as synaptic function; LTP induction and maintenance are, however, normal, likely due to a proportionate reduction in basal and potentiated synaptic transmission. PORCN knockdown also alters AMPAR kinetics, most likely through an altered composition of the complexes, as association with γ8 is reduced. Combined with the subcellular localization of PORCN—mainly at the ER, with only a small portion at the PSD—this suggests that PORCN regulates the early formation of AMPAR complexes at the ER level. Of note, like CPT-1C, FRRS1L, and ABHD6, this role of PORCN is independent of its enzymatic activity [414].

Like ABHD6 and 12, PORCN was identified as part of this early type of AMPAR complexes by Brechet and colleagues [416]. However, the formation of the CPT-1C/FRRS1L/Sac1 complexes in the ER predates the binding of GluA complexes, which is itself required for the subsequent association of PORCN and ABHD6 and 12; this suggests a precise timing in the regulation of the first steps of AMPAR complex formation (Fig. 7). As the constitution of AMPAR complexes at the surface differs between neuronal types, and as ER-specific AMPAR complexes were identified in the hippocampus, future investigation is required to determine whether a similar complex is present in the ER of other neuronal types, if the composition of this ER complex varies according to neuronal types, and if this ER complex influences the composition of the subsequent complexes accompanying AMPARs to the synapse, and thereby its regulation of AMPAR trafficking and function.

## Concluding remarks

AMPA receptor trafficking to, at, and from the synapse has now been established as one of the core mechanisms underlying synaptic plasticity. It is tightly controlled throughout development and adulthood, and dysregulation is not only one of the hallmarks of physiological and pathological aging, but also of many neurological disorders [16]. Over the last two decades, the importance of AMPAR interacting partners in regulating AMPAR trafficking has become increasingly clear—not only because removing or impairing these proteins affects AMPAR trafficking in research models, but also because mutations in AMPAR interactants have been often found in human patients suffering from schizophrenia or autistic spectrum disorders (see, e.g., for reviews [427, 428]). In recent years, a significant headway has been made to understand the roles of these interactants as regulators of AMPAR trafficking; however, much remains shrouded in mystery. While the major role of some of the better known proteins seems well-established—GRIP1 in AMPAR insertion and stabilization, PICK1 in AMPAR internalization and anchorage, and PSD95 in AMPAR synaptic trapping—detailed mechanisms are still unclear; additional tasks have also been uncovered for some interactants, and the functions of the most recent members of AMPAR complexes are still enigmatic. Further intricacy is brought by the dependence of the function of some AMPAR interactants on age, brain region, neuronal population, and even subcellular localization. As these proteins often act in association, such complex roles may also arise from the combination of the different AMPAR interactants available, given their restricted spatio-temporal expression patterns. As if the situation was not convoluted enough, AMPAR interactants—like AMPAR subunits themselves—are also subjected to a variety of posttranslational modifications regulating their function, such as palmitoylation or ubiquitination, and which also need to be integrated into regulatory models of AMPAR trafficking (see for reviews [429, 430]). A major challenge of the upcoming research on AMPAR trafficking will, hence, be to study AMPAR interactants not only in different neuronal types and at different time points, but also to widen the focus from one protein of interest to the combination present in the given neuronal population. Increased collaborations and interdisciplinary research, combining findings from the structural to the behavioral level, but also including data from human patients, are, therefore, required to progress towards a comprehensive picture of AMPAR trafficking.

## Abbreviations

ABHD: a/b-hydrolase domain-containing protein; monoacylglycerol lipase
ABP: AMPAR-binding protein
ADAM: A disintegrin and metalloproteinase
AKAP: A kinase anchoring protein
AMPA(R): α-Amino-3-hydroxy-5-methyl-4-isoxazolepropionic acid (receptor)
AP: Adaptor protein complex
ApoER2: Apolipoprotein E receptor 2
APP: Amyloid precursor protein
Arp: Actin-related protein
ASIC: Acid-sensing ion channel
Atad: ATPase family AAA domain-containing
BAR: Bin-amphiphysin-Rvs
BMP: Bone morphogenetic protein
CACNG: Calcium channel γ subunit
CaM: Calmodulin
CaMK: Calcium/calmodulin-dependent protein kinase
CASK: Calcium/calmodulin-dependent serine protein kinase
CK: Caseine kinase
CKAMP: Cystine-knot AMPAR modulating protein
CNIH: Cornichon homolog protein
CPEB: Cytoplasmic polyadenylation element-binding protein
cpg: Candidate plasticity gene
CPT: Carnitine palmitoyltransferase
DHPG: Dihydroxyphenylglycine
DLG: Discs large homolog
DSP A-D: Dispanin subfamily A–D
EEA: Early endosome antigen
EphB: Erythropoietin-producing human hepatocellular receptor
ER: Endoplasmic reticulum
ERK: Extracellular signal-regulated kinase
FRRS1L: Ferric-chelate reductase 1-like protein
GABA: Aminobutyric acid
GIT: ARF GTPase-activating protein
GK: Guanylate kinase
GPC: Glypican
GPI: Glycosylphosphatidylinositol
GRASP: GRIP1-associated protein
GRIP: Glutamate receptor-interacting protein
GSG1L: Germline-specific gene 1-like
GSK: Glycogen synthase kinase
ICA: Islet cell autoantigen
IgSF: Immunoglobulin superfamily member
JNK: c-Jun-N-terminal pathway
KIBRA: Kidney and brain expressed protein
KIF: Kinesin superfamily
KSR: Kinase suppressor of Ras
LAR-RPTP: Leukocyte common antigen-related receptor protein tyrosine phosphatase
LRP: Low-density lipoprotein receptor-related protein
LRRTM: Leucine-rich repeat transmembrane protein
LTD: Long-term depression
LTP: Long-term potentiation
MAGI: Membrane-associated guanylate kinase, WW and PDZ domain-containing protein
MAGUK: Membrane-associated guanylate kinase
MAP: Microtubule-associated protein
MAPK: Mitogen activated protein kinase
MAP-LC: MAP light chain
Mdm: Mouse double minute
MPP: MAGUK p55 subfamily member
NEEP: Neuron-enriched endosomal protein
NMDA(R): *N*-methyl-d-aspartate (receptor)
NO: Nitric oxide
nPIST: Neuronal isoform of protein interacting specifically with TC-10
NSF: *N*-ethylmaleimide-sensitive fusion protein
PACSIN: Protein kinase C and casein kinase substrate in neurons
Par: Partitioning-defective kinase
PDZ: Postsynaptic density (PSD) 95, *Drosophila* discs large homolog (Dlg) 1, zonula-occludens 1 protein (zo1)
PICK: Protein interacting with C Kinase
PK: Protein kinase
PORCN: Protein-serine O-palmitoleoyltransferase porcupine
PP: Protein phosphatase
PRRT: Proline-rich transmembrane protein
PSD: Postsynaptic density
PTP: Protein tyrosine phosphatase
PV: Parvalbumin
Rap: Ras-related protein
RasGEF: Ras guanine nucleotide exchange factor
Sac: Phophatidylinositide phosphatase Sac
SAP: Synapse-associated protein
Ser: Serine
SH3: Src homology 3
SNAP: Soluble NSF attachment protein
SNARE: Soluble NSF attachment protein receptor
SorCS: Sortilin-related VPS10 domain-containing receptor
*stg*: Stargazer
SynCAM: Synaptic cell adhesion molecule
SynDIG: Synapse differentiation induced gene
TARP: Transmembrane AMPAR regulatory protein
Thr: Threonine
TSPAN: Tetraspanin
TTX: Tetrodotoxin
Tyr: Tyrosine
UPR: Unfolded protein response
